# ABA-mediated responses to water deficit separate grapevine genotypes by their genetic background

**DOI:** 10.1186/s12870-016-0778-4

**Published:** 2016-04-18

**Authors:** Landry Rossdeutsch, Everard Edwards, Sarah J. Cookson, François Barrieu, Gregory A. Gambetta, Serge Delrot, Nathalie Ollat

**Affiliations:** UMR EGFV, ISVV-INRA, 210 chemin de Leysotte, 33882 Villenave d’Ornon, France; CSIRO Agriculture, Private Bag 2, Glen Osmond, SA 5064 Australia; UMR EGFV, ISVV-Bordeaux University, 210 chemin de Leysotte, 33882 Villenave d’Ornon, France; UMR EGFV, ISVV-Bordeaux Sciences-Agro, 210 chemin de Leysotte, 33882 Villenave d’Ornon, France

**Keywords:** Abscisic acid, ABA signalling, Genotypic variability, Grapevine, Roots, Shoot, Transpiration, Water potential, Water-deficit

## Abstract

**Background:**

ABA-mediated processes are involved in plant responses to water deficit, especially the control of stomatal opening. However in grapevine it is not known if these processes participate in the phenotypic variation in drought adaptation existing between genotypes. To elucidate this question, the response to short-term water-deficit was analysed in roots and shoots of nine *Vitis* genotypes differing in their drought adaptation in the field. The transcript abundance of 12 genes involved in ABA biosynthesis, catabolism, and signalling were monitored, together with physiological and metabolic parameters related to ABA and its role in controlling plant transpiration.

**Results:**

Although transpiration and ABA responses were well-conserved among the genotypes, multifactorial analyses separated *Vitis vinifera* varieties and *V. berlandieri* x *V. rupestris* hybrids (all considered drought tolerant) from the other genotypes studied. Generally, *V. vinifera* varieties, followed by *V. berlandieri* x *V. rupestris* hybrids, displayed more pronounced responses to water-deficit in comparison to the other genotypes. However, changes in transcript abundance in roots were more pronounced for *Vitis* hybrids than *V. vinifera* genotypes. Changes in the expression of the cornerstone ABA biosynthetic gene *VviNCED1*, and the ABA transcriptional regulator *VviABF1*, were associated with the response of *V. vinifera* genotypes, while changes in *VviNCED2* abundance were associated with the response of other *Vitis* genotypes. In contrast, the ABA RCAR receptors were not identified as key components of the genotypic variability of water-deficit responses. Interestingly, the expression of *VviSnRK2.*6 (an *AtOST1* ortholog) was constitutively lower in roots and leaves of *V. vinifera* genotypes and higher in roots of *V. berlandieri* x *V. rupestris* hybrids.

**Conclusions:**

This study highlights that *Vitis* genotypes exhibiting different levels of drought adaptation differ in key steps involved in ABA metabolism and signalling; both under well-watered conditions and in response to water-deficit. In addition, it supports that adaptation may be related to various mechanisms related or not to ABA responses.

**Electronic supplementary material:**

The online version of this article (doi:10.1186/s12870-016-0778-4) contains supplementary material, which is available to authorized users.

## Background

*Vitis vinifera* is the major grapevine species grown and is commonly grafted onto rootstocks of other *Vitis* species. The diversity within *Vitis* genus provides a good resource to select from in order to protect against phylloxera and be adapted to various environmental conditions. Among these conditions, water availability is particularly important because of its large influence on fruit yield and quality [1]. Grape growing is common across dry and semi-dry climates and is traditionally non-irrigated [2]. Despite the fact that grapevines are well adapted to dry climates [1], the impact of drought on grape growing may increase in the context of climate change and will lead to changes in viticultural practices and/or the locations suitable for grape growing [3]. Drought negatively impacts grape yields by reducing bud fertility, fruit set and growth [4]. There are large differences in drought tolerance among grapevine genotypes in the field [1] (and references cited therein).

Abscisic acid (ABA) is a stress response and signalling molecule, which plays a central role in the growth, development and adaptation of plants to environmental stresses [5–7]. One of the main functions of ABA is to regulate plant water balance and osmotic stress tolerance. ABA mediates numerous responses to drought, including stomatal closure and control of water loss from the plant [8–10]. Grapevines were among the first species in which a direct role of ABA in stomatal closure was demonstrated [11]. Subsequently, ABA was shown to be associated with water-deficit responses at the root, leaf, shoot and fruit levels [12]. Genotypic differences in leaf ABA concentration have been known for many decades [13, 14]. Among *Vitis* genotypes, differences in stomatal sensitivity to drought have been associated with ABA concentration in xylem sap or leaves [15], and there is variability in stomatal sensitivity to ABA [16–18].

Under drought, ABA is synthesized in roots [19], shoots [9] and leaves [20]. ABA synthesis in roots and its transport to the leaves has been considered the main signalling pathway transducing soil water status [21–23] because of the correlation between stomatal conductance and ABA concentration in xylem sap [24–26]. In addition, hydraulic signals could modulate stomatal closure either directly, and/or via ABA production in the leaf [9, 27]. Furthermore, recent studies suggest that the extent to which stomatal conductance is controlled by either hydraulic signals, ABA or their interaction could be associated with genetic differences in responses to drought [27, 28]. In grafted plants including grapevine, it was shown that rootstocks affect both ABA concentration [ABA] in xylem sap and stomatal sensitivity to drought [22, 29, 30].

ABA biosynthesis begins in plastids with the cleavage of a C40 carotenoid precursor that is further epoxidized to 9-cis-violaxanthin. Then 9-cis-epoxycarotenoid dioxygenase (NCED) catalyses the oxidative cleavage of 9-cis-violaxanthin to form xanthonin [31]. These products enter the cytosol where a dehydrogenase/reductase and an aldehyde oxidase convert xanthonin into ABA. The vast majority of ABA is catabolized to its inactive form by an ABA 8′-hydroxylase. The spontaneous cyclization of hydroxylated ABA results in the production of phaseic acid (PA) which is further reduced to dihydrophaseic acid (DPA) [5]. In grapevine it was shown that the expression of NCED genes in both leaves and roots is well correlated with [ABA] in xylem sap and stomatal opening [29, 30]. In addition, changes in ABA catabolism near its site of action could optimize gas exchange to the local leaf environment as the expression of ABA catabolic genes in leaves appear to change in response to vapour pressure deficit (VPD) [30].

The ABA signalling pathway involves a cascade of receptors, phosphatases, kinases and transcription factors (TFs), which have been well characterized [5, 6, 32–35]. The key components of this system are the protein receptor complex PYR/PYL/RCAR (PYRABACTIN RESISTANCE1)/(PYR1-LIKE)/(REGULATORY COMPONENTS OF ABA RECEPTORS), PP2Cs (PROTEIN PHOSPHATASE 2C) and SnRK2s (SUCROSE NON-FERMENTING-RELATED KINASE 2). In the absence of ABA, PP2Cs inactivate SnRK2s kinases by physical interaction and direct de-phosphorylation. The binding of ABA to PYR/PYL/RCAR leads to a conformational change in the receptor enabling its interaction with PP2Cs and thereby activating the SnRK2s. The SnRK2s released from PP2C inhibition are then able to activate (via phosphorylation) downstream transcription factors (TF) and ABA-responsive element binding factors (ABFs or AREBs), leading to the induction of ABA-responsive genes [5, 6, 34, 36]. Most of the components of the ABA signal transduction pathway have been identified in the *V. vinifera* genome [37–39]. The grapevine genome encodes at least seven PYR/PYL/RCAR ABA receptors, six PP2Cs, six SnRK2 kinases and several ABA-related TFs.

Under abiotic stress conditions, including water-deficit, most of the ABA biosynthetic and catabolic genes are transcriptionally induced [34, 40–42]. In contrast, the transcriptional regulation of ABA signalling pathway genes is more varied. For example, some genes encoding PYR/PYL/RCAR receptors are repressed in both leaves and roots by abiotic or biotic stresses, or ABA treatments, but others are unaffected or transiently induced [34, 41, 43, 44]. In barley, the expression of some PYR/PYL/RCAR genes was unchanged after 4 days of water-deficit, but reduced after 12 days of water-deficit, indicating that the duration of the treatment affects the response [41]. PP2C genes are generally induced under stress conditions [34, 38, 39, 41, 43–45]. In Arabidopsis, the induction of SnRK2 gene expression depends on the member of the gene family and stress type [34], and the expression of the transcriptional regulators of ABA signalling (e.g. ABFs) increases in response to ABA and water-deficit [34, 36, 46].

The aim of this work was to determine whether the commonly observed differences in drought adaptation of nine grapevine genotypes (defined in Table 1) were associated with differences in ABA metabolism and the expression of genes involved in ABA biosynthesis, catabolism and transduction pathways. Plant and soil water status, plant transpiration, the content of ABA and its catabolites, and the transcript abundance of 12 genes involved in ABA metabolism and signalling (previously described in the literature in grapevine, [30, 38, 39, 47]) were characterized in response to withheld irrigation in roots and leaves. These data were used to characterize the variability existing among *Vitis* genotypes, especially the drought tolerant ones, in terms of the contribution of ABA to water-deficit responses.

**Table 1:** Parentage and drought sensitivity of the genotypes studied [62, 63]

Genotypes (clone number)	Usual name	Parentage	Drought sensitivity
Riparia Gloire de Montpellier (1030)	RGM	*V. riparia Michaux*	Highly sensitive
Millardet et de Grasset 101-14 (1043)	101-14Mgt	*V. riparia* x *V. rupestris*	Sensitive
Téléki-Fuhr Selection Oppenheim n°4 (762)	SO4	*V. riparia* x *V. berlandieri*	Sensitive
Couderc 161-49 (197)	161-49C	*V. riparia* x *V. berlandieri*	Medium
Millardet et de Grasset 41B (194)	41B	*V. vinifera L.* x *V. berlandieri*	Medium
Richter 110 (756)	110R	*V. berlandieri* x *V. rupestris* Martin	Tolerant
Ruggeri 140 (101)	140Ru	*V. berlandieri* x *V. rupestris* du Lot	Tolerant
Syrah (524)	Syrah	*V. vinifera*	Tolerant
Grenache (136)	Grenache	*V. vinifera*	Drought Avoiding

## Results

### Genotype-specific transpiration responses to water-deficit

**Fig. 1:**
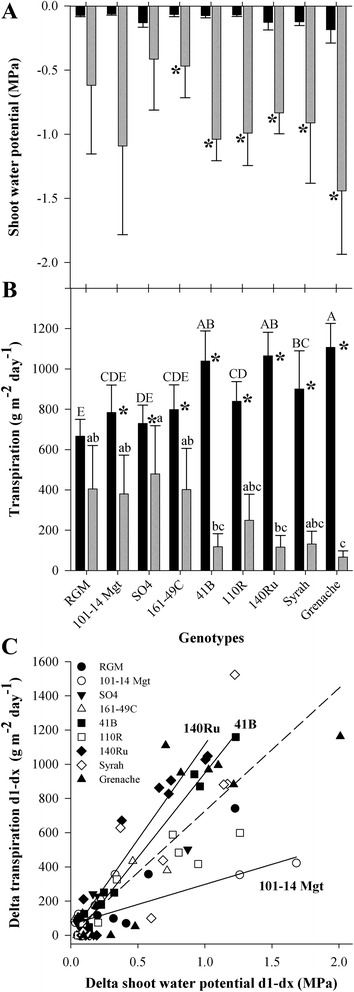
Physiological responses of nine grapevine genotypes to water-deficit. Shoot water potential (**a**) and transpiration (**b**) 1 day (black bars) and 4 days (grey bars) after withholding irrigation. For A and B, bars represent mean ± standard deviation (*n* = 3) and asterisks show significant water-deficit effect (Kruskall Wallis, *p*-value < 0.05). For B, values among genotypes with the same letter are not statistical different (day 1 and day 4 analysed separately with an ANOVA on ranks, *p*-value < 0.05). The relationship between the changes in transpiration and shoot water potential (**c**), key to symbols: RGM, filled circle; 101-14Mgt, open circle; SO4, inversed filled triangle; 161-49C, open triangle; 41B, filled square; 110R, open square; 140Ru, filled diamond; Syrah, open diamond; Grenache, filled triangle. The dashed line shows the global linear regression for all nine genotypes, solid lines show those genotypes with a significantly different relationship from the global linear regression (Fischer-Snedecor test; *p* < 0.05)

Four days after withholding irrigation, average pre-dawn shoot water potential was significantly reduced in all genotypes with the exception of SO4 (Fig. 1a). Average pre-dawn water potentials ranged between -0.4 to -1.5 MPa representing moderate to severe levels of water-deficit. The genotype effect was not statistically significant (Fig. 1a). Water potential was maintained until soil water content reached 0.04 g H_2_O g^-1^ of dry soil, and then it decreased (Additional file 1).

Plant transpiration was significantly reduced by water-deficit in all genotypes except RGM (Fig. 1b). A significant genotype effect was observed at days 1 and 4. The response of the genotypes can be separated into two groups: RGM, 101-14Mgt, SO4 and 161-49C were characterized by relatively low transpiration at day 1 and higher transpiration than other genotypes at day 4, whereas 41B, 140Ru and Grenache were characterized by relatively high transpiration at day 1 and low transpiration at day 4, with 110R and Syrah being intermediate. Transpiration per plant was reduced in response to decreasing water potential (Fig. 1c). Statistical comparison of the slopes between genotype specific regressions and general regressions (including all the genotypes) revealed that, in response to decreasing shoot water potential, 140Ru and 41B decreased significantly more their transpiration and 101-14Mgt decreased it significantly less than the bulk of genotypes.

### Genotype-specific differences in ABA metabolism for non-stressed and water-stressed plants

**Fig. 2:**
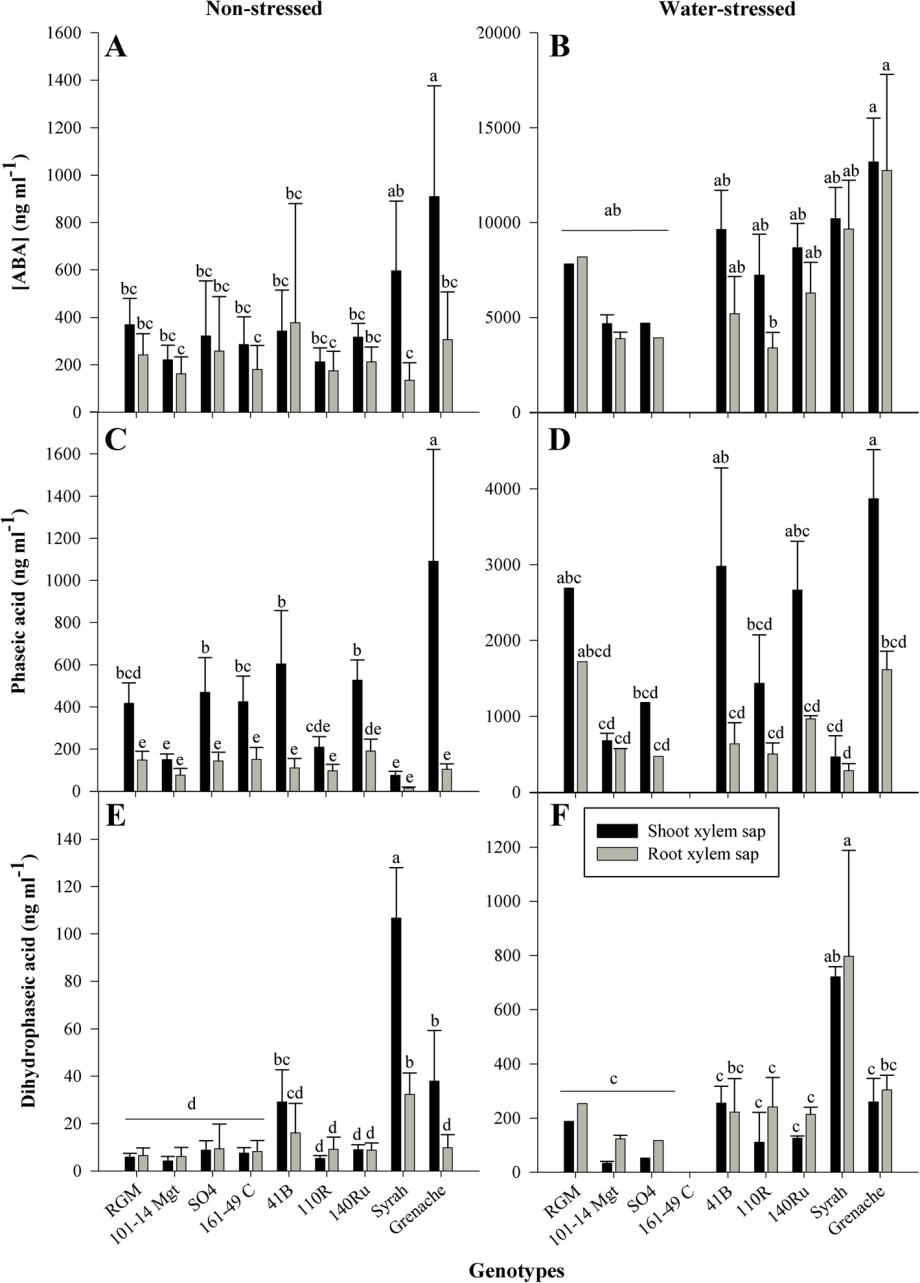
Concentration in ABA, PA and DPA in shoot and root xylem sap (ng/ml). Mean and standard deviation of abscisic acid (ABA; **a** & **b**), phaseic acid (PA; **c** & **d**) and dihydrophaseic (DPA; **e** & **f**) for non-stressed (water potential > -0.2 MPa; **a**, **c** & **e**) and water-stressed (water potential < -0.8 Mpa; **b**, **d** & **f**) plants. Values among genotypes with the same letter are not statistically different (Tukey-HSD) (*n* = 1–10)

ABA concentration ([ABA]) and the concentration of its degradation products, [PA] and [DPA], were determined in the xylem sap collected from root and shoot parts. Globally, [ABA], [PA] and [DPA] were strongly correlated between root and shoot xylem sap (Additional file 2) and both [PA] and [DPA] were strongly correlated with [ABA] (Additional file 3). Average [ABA], [PA] and [DPA] in shoot and root xylem sap for the different genotypes for non-stressed and water-stressed plants are presented in Fig. 2. Concentrations were significantly affected by genotype. Water-stressed Grenache had the highest [ABA] in shoot and root xylem sap, but the only significant difference was with 110R in roots. Grenache had significantly the highest [PA] in the shoot xylem sap in comparison with all the non-stressed genotypes and in comparison with water-stressed Syrah, 110R, SO4 and 101-14Mgt. [PA] was the highest in root xylem sap of water-stressed Grenache and RGM, but not significantly in comparison with the other genotypes. Syrah was characterized by significantly higher [DPA], regardless of plant part and water status. In shoot xylem sap of water-stressed plants, the differences with Syrah were significant for 140Ru, 110R, SO4 and 101-14Mgt.

### Changes of [ABA], [PA], and [DPA] with plant water status

**Fig. 3:**
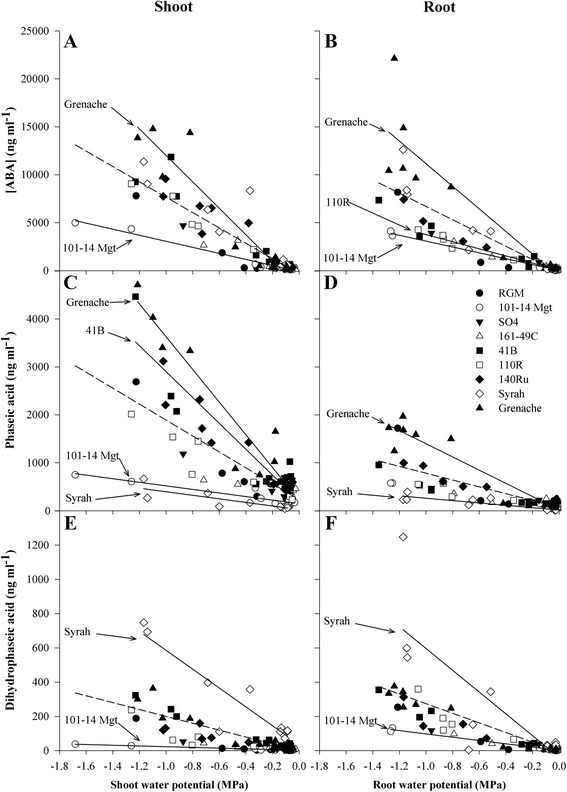
Relationships between water potential and concentration of ABA, PA, DPA in root and shoot xylem sap. ABA (**a**, **b**), phaseic acid (**c**, **d**) and dihydroxyphaseic acid (**d**, **e**) in root (**a**, **c** & **e**) and shoot (**b**, **d** & **f**) xylem sap during a four day water-deficit treatment in nine grapevine genotypes (key to symbols as shown for Fig. 1c) (*n* = 12). The dashed line shows the global linear regression for all nine genotypes, solid lines show those genotypes with a significantly different relationship from the global linear regression (Fischer-Snedecor test; *p* < 0.05). The slopes of the different regressions estimate the accumulation plasticity of the various genotypes for the different compounds. Comparisons between the slopes of general regressions obtained for shoot and root xylem sap were made using a Fischer-Snedecor-test (ABA: F = 1.76, *p* < 0.05; PA: F = 37.7, *p* < 0.001: DPA: F = 3.57, *p* < 0.05)

The [ABA], [PA] and [DPA] increased significantly in the xylem sap of the shoots and roots while water potential decreased for all genotypes. The slope of the response curve to water potential is an estimation of the accumulation capacity. In order to compare the accumulation capacity of individual genotypes to the average accumulation capacity, the general regressions and the genotype-specific regressions, significantly different from the general regressions, are presented in Fig. 3.

The statistical comparisons of slopes between general regressions for shoot and root xylem sap indicate that the general responses of [ABA], [PA] and [DPA] to plant water potential were significantly different between the shoot and root xylem sap (Fig. 3, *p* < 0.05, *p* < 0.001, *p* < 0.05 respectively). The increase of concentration was higher in shoot xylem sap in comparison to root xylem sap for ABA and PA, and the opposite for DPA (Additional file 2). As plant water potential became more negative, Grenache displayed the greatest increase in [ABA] and [PA]. 101-14Mgt showed the smallest [ABA] increase in both shoot and root xylem sap. 110R was characterized by a significantly smaller increase of [ABA] only in root xylem sap. Syrah showed the smallest increase in [PA] in both plant parts. Additional significant differences in [PA] between genotypes were found in shoot xylem sap for 41B and 101-14Mgt. In comparison to the bulk of the genotypes, 41B and 101-14Mgt were characterized by a greater and smaller increase in [PA] respectively. For [DPA], Syrah displayed a more pronounced increase with decreasing water potential, while the opposite was observed for 101-14Mgt.

**Fig. 4:**
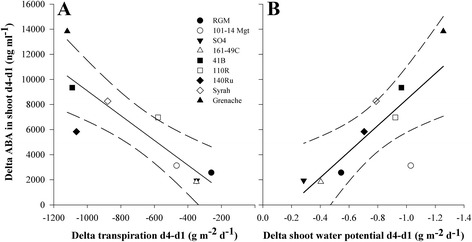
Relationship between ABA concentration changes and plant water status. Plots of the changes from day 1 to day 4 after withholding irrigation in abscisic acid (ABA) concentration in the shoot sap and transpiration (**a**) and shoot water potential (**b**) for nine grapevine genotypes (key to symbols as shown for Fig. 1c). Each point represents difference between means at day 4 and at day 1 (*n* = 3). The black lines show the global linear regression for all nine genotypes, dashed black lines show the 95 % interval of confidences for the regressions

From day 1 to day 4 without irrigation, the changes in [ABA] in shoot sap were highly correlated to changes in transpiration (Fig. 4a, *R*^2^ = 0.86, *p* < 0.01) and pre-dawn shoot water potential (Fig. 4b, *R*^2^ = 0.80, *p* < 0.01) across all the genotypes. Similar results were obtained for roots (data not shown). Several genotypes were situated outside of the confidence intervals of the regressions. Grenache had the largest difference in both [ABA] and transpiration, while 140Ru had much smaller differences in [ABA] with similarly large reduction in transpiration. The genotype 101-14Mgt was also an outlier in the relationship between change in [ABA] and shoot water potential, showing a much smaller increase of [ABA] in relation to the decrease in pre-dawn shoot water potential.

### Effects of water-deficit on transcript abundance of ABA related genes

**Fig. 5:**
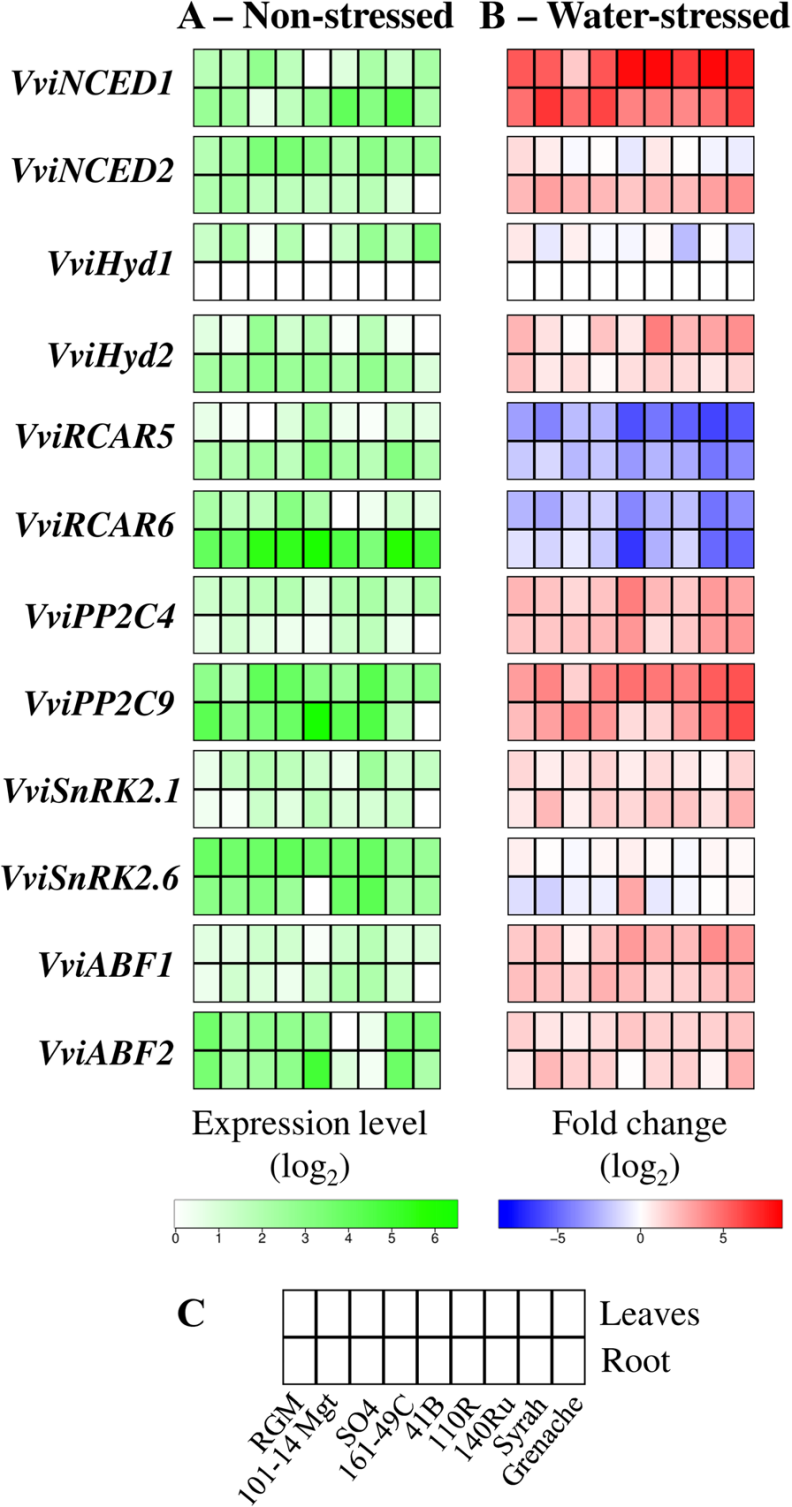
Heatmaps of the abundance of transcripts for studied genes and their variations with water deficit. The abundance of transcripts for the genes associated with abscisic acid was recorded in the leaves and roots of nine grapevine genotypes during a water-deficit treatment. Transcript abundance at day 1 after withholding irrigation (non-stressed plants) (**a**), green shade indicates the level of expression relative to the lowest value (*n* = 3). Transcript abundance changes from day 1 to day 4 after withholding irrigation (water-stressed plants) (**b**), the blue and red shades indicate the extent of gene repression and induction respectively (*n* = 3). Blocks of squares show the level of gene expression in the leaves and roots of nine different grapevine genotypes (**c**) for each gene studied

The transcript abundance of 12 ABA-related genes was studied in non-stressed (Fig. 5a) and water-stressed plants (Fig. 5b). The heat map for non-stressed plants presents the level of expression normalised for each gene by the lowest expression either in leaves or roots. The heat map for water-stressed plants presents the ratio of the average expression at day 4 to the average expression at day 1 for each genotype and tissue. Results are expressed in log_2_ (Fold-change relative to day 1 expression). Average expression data per genotype and water treatment, and results of ANOVA analyses are given in Additional files 4, 5 and 6.

In non-stressed plants, the abundance of all transcripts was modified significantly by the plant tissue and by the genotype, with the exception of *VviABF1* for the plant tissue and *VviNCED1* for the genotype (Fig. 5a and Additional file 4). ANOVA analysis shows that a significant higher abundance of transcripts was recorded in leaves of *VviNCED2, VviHyd1, VviPP2C4, VviSnRK2.1* and *VviSnRK2.6* while the abundance was higher in roots for *VviNCED1, VviHyd2, VviRCAR5, VviRCAR6* and *VviABF2.* Among the genotypes, Grenache was characterized by the lowest abundance of transcripts for *VviNCED2, VviPP2C4, VviPP2C9, VviSnRK2.1* and *VviABF1* in roots*.* This genotype, as well as 140Ru, presented a high abundance of transcripts in leaves for *VviHyd1* and *VviPP2C4*. 140Ru presented the higher abundance of *VviSnRK2.1* in leaves, and together with 110R the higher abundance of *VviABF1* in roots and the lower abundance of *VviABF2* both in leaves and roots. Finally 41B was characterized by a low abundance of transcripts of *VviHyd1* and *VviABF1* in leaves, *VviSnRK2.6* in roots, and the highest abundance for *VviABF2* in roots.

The extent to which transcript abundance was modified in water-stressed plants is presented in Fig. 5b. According to ANOVA analysis, water stress significantly affected the abundance of all transcripts, excepted *VviNCED2* in the leaves*,* and *VviHyd1* and *VviSnRK2.6* in the roots. Genotypes significantly affected the abundance of all transcripts in leaves. In the roots, the abundance of transcripts was significantly affected by genotypes for *VviNCED2*, *VviHyd1, VviRCAR6*, *VviPP2C9, VviSnRK2.6* and *VviABF2* in the roots (Additional files 5 and 6).

The abundance of the transcripts *VviNCED1*, *VviHyd2*, *VviPP2C4*, *VviPP2C9, VviSnRK2.1 VviABF1* and *VviABF2* were significantly increased, both in the leaves and the roots for all genotypes (log2 fold change < -2 or > 2 or *p* < 0.05). Generally, the abundance of *VviRCAR5* and *VviRCAR6* decreased in the leaves and the roots. For *VviSnRK2.6*, log2 Fold change was below two in the leaves, but ANOVA analysis detected a significant increase, whereas in the roots, no significant change was detected although the ratio of expression was above two for 41B. Grenache displayed the highest increase in transcript abundance in leaves for *VviHyd2*, *VviABF1*, in roots for *VviNCED2*, and in both leaves and roots for *VviPP2C4*, *VviPP2C9*, *VviSnRK2*.1 and *VviABF2*. This genotype presented also a more pronounced decrease for *VviRCAR5* and *VviRCAR6* in leaves and roots. Syrah and 41B presented the same pattern as Grenache for *VviRCAR5*, *VviRCAR6* and *VviPP2C4* both in leaves and roots. In addition Syrah presented the same pattern as Grenache for *VviNCED2* in roots, and *VviPP2C9* both in leaves and roots, and 41B for *VviABF2* in leaves. Finally 110R and 140Ru displayed also a pronounced decrease of *VviRCAR5* both in leaves and roots. Both genotypes had a common response as Grenache for *VviHyd2* and *VviHyd1* in leaves.

The transcript abundances of many of the genes studied were correlated with one another (Additional file 7). *VviNCED1* was highly correlated with *VviPP2C4* in leaves and *VviABF1* in the leaves and roots. *VviPP2C4* was highly positively correlated with *VviNCED1*, *VviABF1* and *VviPP2C9* in leaves, but negatively with *VviRCAR5* and *VviRCAR6* both in the leaves and roots. The abundance of *VviRCAR5* and *VviRCAR6*, both in leaves and roots, were positively correlated with each other, and negatively correlated with *VviPP2C4* and *VviABF1* in leaves.

### Multi-factorial analyses of genotype-specific responses to water-deficit

**Fig. 6:**
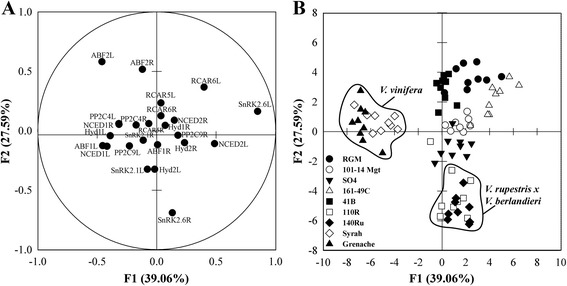
Factorial discriminant analysis of the transcript abundance with the genotype as qualitative sorting variable. The abundance of transcripts for12 genes associated with ABA was recorded 1, 3 and 4 days after withholding irrigation in nine grapevine genotypes. The distribution of variables (**a**) and individual observations (**b**) on factors F1 and F2. For A, transcript abundance of each gene is presented in leaves (L) and root tips (R). For B, key to symbols as shown in Fig. 1c

A discriminant analysis (Fig. 6) was conducted on transcript abundance with genotype as qualitative sorting variable. The first two discriminant functions of this analysis, F1 and F2, explained 39.1 and 27.6 % of total variability, respectively (Fig. 6a). F1 was positively correlated with the abundance of *VviSnRK2.6*, *VviNCED2* and *VviRCAR6*, and negatively correlated with the abundance of *VviNCED1*, *VviHyd1*, *VviABF1* and *VviABF2* in leaves (Fig. 6a, Additional file 8). F2 was positively correlated with the abundance of *VviABF2* and *VviRCAR6* in leaves and *VviABF2* in roots, and negatively correlated with the abundance of *VviSnRK2.6* in roots (Fig. 6a). The score plot of observations on the plan defined by F1 and F2 shows that the genotypes are well discriminated (Fig. 6b). Syrah and Grenache are both discriminated along the negative side of F1, and not along F2. 110R and 140Ru are discriminated along the negative side of F2, and not along F1. The other genotypes were mainly distributed along the F2 axis with SO4 on the negative side, 41B, RGM and 161-49C on the positive side. RGM and 161-49C were also distributed positively along F1.

**Fig. 7:**
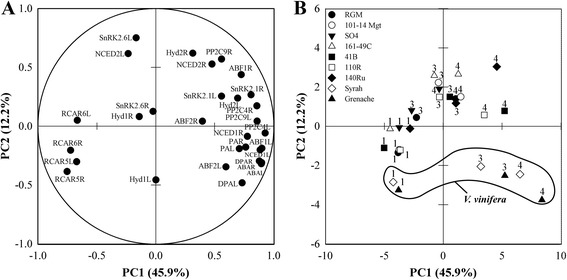
Principal component analysis of physiological and transcript abundance data. Plots for variable contribution to each principal component (**a**) and projection of individual observations (**b**) on PC1 and PC2. For A, mean of expression of each gene is presented in leaves (L) and root tips (R) and mean of abscisic acid (ABA), phaseic acid (PA) and dihydroxyphaseic acid (DPA) is presented in shoot (S) and root (R) xylem sap. For B, key to symbols as shown in Fig. 1c, numbers indicate the number of days of withheld irrigation

Finally, a principle component analysis was done on the average of all raw data per genotype and day of sampling. The first two components, PC1 and PC2, explained 63 % of total variability (Fig. 7). The abundance of transcripts of most genes, as well as all physiological variables, were highly correlated to PC1, except for *VviSnRK2.6* and *VviNCED2* in the leaves, which were highly correlated to PC2 (Fig. 7a). [ABA], [PA] and [DPA] cluster tightly with the expression of *VviNCED1* in both tissues, and with *VviABF1*, *VviABF2* and *VviPP2C4* in the leaves (Fig. 7a, Additional file 8). The score plot of individual observations on the plan defined by the first two main components shows that PC1 and PC2 are mainly described by the water status and genotype effects respectively. Under water-stress, all genotypes shifted towards the positive side of PC1 with 140Ru, 110R and 41B located in an intermediate position along PC1, between Syrah and Grenache and the other genotypes studied. Some variability can also be observed between genotypes along PC1 for their response at 3 days of withheld irrigation. In addition water-stressed Syrah and Grenache (Fig. 7b) remained on the negative part of PC2 while the other genotypes moved to the positive part of this component.

## Discussion

The nine genotypes from different *Vitis* backgrounds studied here displayed common and specific responses to short-term water-deficit in terms of plant water status, ABA metabolite concentration in xylem sap and transcriptional regulation of some genes associated with ABA biosynthesis/catabolism and signal transduction pathways.

### Responses to water-deficit are common to the genotypes studied

All genotypes exhibited typical physiological responses to water-deficit [18, 29, 30, 48]. Soil water content pre-dawn root and stem water potential, and transpiration were significantly reduced. The decrease in daily transpiration was linearly, and positively, correlated with the change in pre-dawn stem water potential. ABA accumulated under water-deficit and the range of [ABA] in stem xylem sap was similar to previous observations for grapevine. [ABA], [PA] and [DPA] were highly correlated, among themselves, and the accumulation of these 3 compounds was quantitatively related to plant water status [19, 49].

Among the three putative homologues of NCED identified in grapevine [50, 51], *VviNCED1* and *VviNCED2* are considered as the two main genes associated with ABA synthesis in response to plant water status [15, 30, 47]. In the present work, *VviNCED1* transcript abundance was highly increased in water-stressed plants while *VviNCED2,* already high in non-stressed plants, was further increased by water-deficit in the roots only. In water-stressed roots, both *VviNCEDs* are associated with increases in [ABA], in agreement with Speirs et al. [30]. The absence of any significant change in *VviNCED2* abundance in water-stressed leaves supports the findings of Soar et al. [47], where *VviNCED2* expression level was shown to be more related to leaf age.

Among the different ABA catabolism pathways, the 8′-hydroxylation is considered as the predominant one [40]. In the present study, the abundance *VviABA8′OH-1* (*VviHyd1*) was not affected by water-deficit (in agreement with Speirs et al. [30]) while the abundance of *VviABA8′OH-2* (*VviHyd2)* transcripts was significantly increased to a larger extent in leaves where it was highly correlated with [ABA], [PA] and [DPA]. Speirs et al. [30] suggested that ABA catabolism in leaves could adjust gas exchanges to VPD; our data support that it also responds to soil water status.

The abundance of *VviNCED2* and *VviHyd1* transcripts were significantly higher in the leaves than in the roots of non-stressed plants, and the abundance of *VviHyd2* was more than two-fold greater in the leaves than in the roots of water-stressed plants. This suggests an important contribution of leaves to ABA biosynthesis and catabolism. Consequently the higher concentrations of [ABA], [PA] and [DPA] in shoot xylem sap in comparison to root xylem sap probably result from root synthetized ABA and local metabolism in leaves [20, 52].

Various PYR/PYL/RCAR members have specialized functions that could be associated with differences between short- and long-term water-deficit responses [41]. In the current study, the abundance of *VviRCAR5* and *VviRCAR6* transcripts, which are the predominantly expressed isogenes identified in the grapevine genome, was reduced by water-deficit. *VviPP2C4* and *VviPP2C9*, as the main interactors with *VviRCARs* [38], were expressed in leaves and roots of non-stressed plants for all genotypes and their abundance was increased in water-stressed plants. The expression pattern of these genes is consistent with studies across multiple species [34, 38, 41, 43–45].

SnRK2 proteins belong to a family of plant-specific serine/threonine kinases that are involved in abiotic and ABA responses [6]. From the *SnRK2* genes identified in the grapevine genome [39], the abundance of *VviSnRK2.1* was increased by water deficit in leaves and roots, while *VviSnRK2.6* was not significantly modified in the roots supporting a similar response as reported for *Arabidopsis SnRK* genes [34].

*VviABF1* and *VviABF2* are orthologs of *AtAREB1/ABF2* [39]. This transcription factor is one of the master elements that regulate ABRE-dependant signalling involved in water-deficit tolerance in vegetative tissues [36, 53]. In the present study, the abundance of both *VviABFs* was increased by water-deficit, but not with organ specificity as reported previously for a dehydration stress [39].

The strong correlations observed for the expression of *VviPP2C4* and *VviABF1* with the expression of most other genes studied here suggest that these two genes could play a central role in the ABA signalling in response to water-deficit in grapevine. Indeed it was shown for Arabidopsis, that plants mutated for AREB/ABF TFs or PP2C genes displayed modifications of sensitivity to ABA and of tolerance to water-deficit [36, 46, 54, 55].

### Genotype-specific responses are associated with their genetic background

The genotypes studied here significantly affected most physiological parameters and gene expression profiles, both in non-stressed and water-stressed plants. Our study provides new knowledge about the mechanisms involved in the intraspecific and interspecific phenotypic diversity reported for water-deficit responses in grapevine [1, 4, 56].

Syrah and Grenache (the *V. vinifera* varieties) were clearly separated from 140Ru and 110R (the *V. berlandieri* x *V. rupestris* hybrids), and from the other genotypes, using a factorial discriminant analysis of the transcript abundance of 12 genes related to ABA in non-stressed and water-stressed plants. The abundance of *VviNCED2, VviSnRK2.6*, *VviABF1* and *2* in leaves were the most discriminant variables separating *V. vinifera* from the other genotypes, while *VviABF2* in leaves, *VviSnRK2.6* and *VviABF2* in roots were the most discriminant variables separating *V. berlandieri* x *V. rupestris* hybrids from the other genotypes. The abundance of *VviNCED2* in leaves and *VviSnRK2.6* in roots was not affected by the water-deficit, indicating a constitutive differential expression of these genes between genotypes. OPEN STOMATA 1 (OST1/At4g33950), the Arabidopsis ortholog of *VviSnRK2.6*, is involved in the regulation of anion and potassium channels, and aquaporin activity in guard cells [57, 58]. Its function in roots has not been investigated, but its role in guard cells may suggest that it participates in the control of ion and/or water transport.

**Fig. 8:**
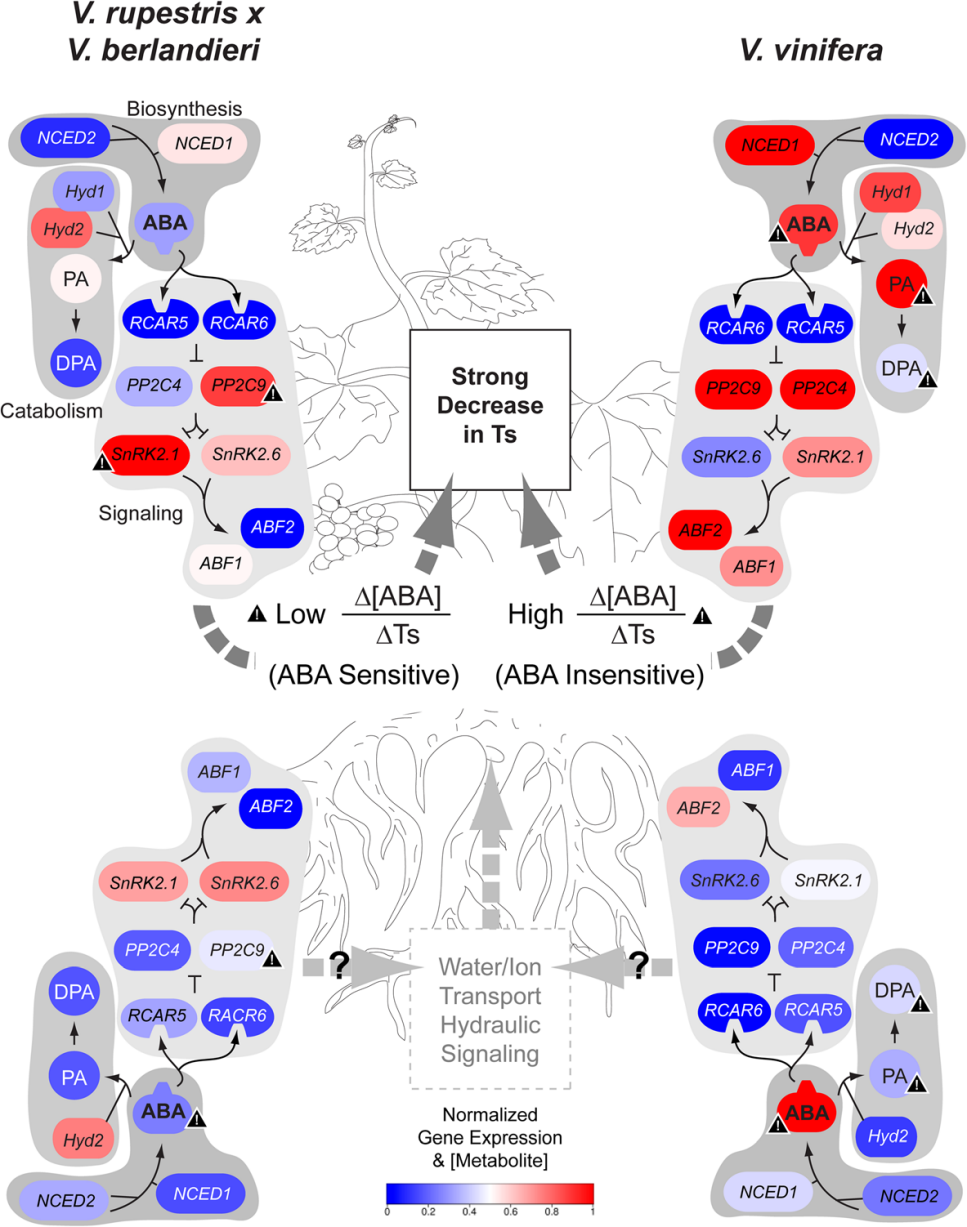
Summarized view of the responses recorded in the experiment for the literature-based tolerant *Vitis* genotypes. ABA-related gene expression, metabolite concentration and transpiration sensitivity to ABA after 4 days of withholding irrigation are illustrated for the genotypes defined in literature as drought tolerant i.e. *V. berlandieri* x *V. rupestris* hybrids (*left*) and *V. vinifera* (*right*). Colours indicate genes expression values and ABA-related metabolite concentration for 140Ru and Grenache scaled between the lowest and the highest values, 4 days after withholding irrigation for all genotypes and tissues. Warning symbols indicate the intra-group variability when it is significant between 140Ru and 110R on one side and between Grenache and Syrah on the other side, according to Fig. 2 & 3a and Additional files 5 & 6

The *V. vinifera* genotypes displayed more pronounced transcriptional responses to the water-deficit treatment than the other genotypes, followed by the *V. berlandieri* x *V. rupestris* hybrids and 41B (a *V. berlandieri* x *V. vinifera* hybrid). These changes are summarized in Fig. 8. The response of *V. vinifera* genotypes to water-deficit was mainly associated with changes in abundance of *VviNCED1* in leaves and roots, and *VviHyd*s and *VviABF*s in leaves. For *V. berlandieri* x *V. rupestris* hybrids and 41B, the intermediate response was associated with the abundance of *VviNCED2* and *VviSnRK2.6* in leaves and*, VviNCED2, VviHyd2, VviPP2C9,* and *VviABF1* in roots. Own rooted *V. vinifera* are considered to better tolerate drought than when grafted on American hybrids [59]. This high drought tolerance could be associated with the ability to regulate the expression of genes that control ABA responses in leaves observed in the present study. In *V. berlandieri* x *V rupestris* hybrids and 41B, which are characterized as drought tolerant rootstocks [13, 56], the response appears to have a relatively stronger root component. The ABA receptors, *VviRCAR5* and *VviRCAR6*, were not identified as key component of the variability of water-deficit responses between the genotypes. The responses of ABA concentration and transpiration to plant water potential were also more pronounced for some of these tolerant genotypes such as Grenache, 140Ru and 41B.

Although the genotypes could be grouped according to their genetic background, some within-groups variability was observed (Fig. 8). For example, among *V. vinifera* varieties, Grenache was characterized by the highest [ABA] in stem xylem sap, significantly higher expression of *VviNCED1* in leaves, and significantly steeper slopes for the relationships between [ABA] and [PA] with plant water status. The higher ratio of delta [ABA] to delta transpiration in Grenache confirms its lower sensitivity to ABA [17]. Grenache is traditionally referred to as a near-isohydric variety, reducing stomatal conductance and leaf transpiration more rapidly in order to avoid a drop in leaf water potential [1, 15, 24, 60]. This link between a lower sensitivity to ABA and higher sensitivity to VPD has been suggested in other studies for Grenache [15]. Among the *V. berlandieri* x V*. rupestris* hybrids, both considered as drought tolerant, 140Ru did not differ from the bulk of genotypes for ABA accumulation capacity, but its transpiration was more reduced for a given [ABA] indicating a higher sensitivity to ABA. On the contrary, 110R displayed a lower accumulation capacity of ABA and its sensitivity to ABA was not different from the bulk of genotypes.

## Conclusions

Despite the observation that global ABA responses to water-deficit are maintained between model species and *Vitis* genotypes, this study shows that several aspects of the ABA metabolism and signalling pathways allow the segregation of the nine genotypes studied according to their genetic background and their drought tolerance level. *V.vinifera* genotypes, and among them Grenache, displayed very specific responses in comparison to the *non-vinifera* genotypes. Our results support that ABA contributes to the genetic control of water-deficit responses in grapevine. Indeed enhancing ABA production and homeostasis lead to improved drought tolerance under long-term stress conditions or at adult stages in several species [41, 55]; and in grapevine, several genes involved in ABA metabolism and signal transduction pathway are located in the confidence interval of QTLs controlling rootstock responses to water deficit [61].

An absolute relationship between high ABA production capacity and known drought tolerance in the field was not established, supporting that drought tolerance could be acquired through different mechanisms [56]. Responses to water deficit were mainly associated with changes in *VviNCED1* and *VviABF1* abundance in *V. vinifera* genotypes which are drought tolerant, while changes in *VviNCED2* abundance was involved for other *Vitis* genotypes. In addition the expression of *VviSnRK2.6* (an AtOST1 ortholog) was constitutively higher in roots of the drought tolerant *V. berlandieri* x *V. rupestris* hybrids. The contribution of these genes to the control of the genetic variability for drought adaptation should be further checked by other approaches such as genetic mapping and functional analysis for *VviSnRK2.6* in roots.

## Methods

### Plant material and water-deficit treatments

The responses of nine grapevine genotypes to water-deficit were analysed; the genotypes selected were commercial inter-specific hybrids and two *V. vinifera* varieties with known differences in response to drought (Table 1) [62, 63]. Hardwoods were obtained from the Aude’s Chamber of Agriculture, France except for 101-14Mgt and 140Ru hardwoods which were obtained respectively from Amblevert and ENTAV nurseries, Gironde, Hérault, France. Hardwood was stored in a cold chamber (4 °C) during the winter, and after one-night of rehydration in water at 25 °C, single-node cuttings were prepared and planted in perforated plastic bags in 0.8 L pots filled with exactly 600 g of dry sand and grown in a greenhouse. Plants were watered with standard nutrient solution [64] and shoots were trained to a single stem until they reached 15 fully expanded leaves. The plants were then transferred to a growth chamber on a turntable with a day/night temperature of 25 °C/19 °C and a VPD of 1.27kPa/0.11kPa. The average photosynthetic flux density at the canopy level was around 400 μmol m^-2^ sec^-1^ during a 16 h light cycle.

In order to avoid a too large variability in the rate of decrease in soil water content, leaf area was normalized to approximately 400 cm^2^ by removing entire leaves from the base of the stem three days before the beginning of the experiment. It was assumed that the plants had recovered from the stress of leaf removal when the experiment started and that the main differences recorded during the short term water deficit were mainly associated to water status. Leaf area was estimated from the relationship between leaf area (measured with a planimeter (Li 3100, Li-COR Biosciences, Lincoln, NE, USA)) and leaf main vein length for each genotype in a separate experiment (data not shown). Leaf normalization resulted in a coefficient of variation of 3.4 % across genotypes (Additional file 9). Nevertheless, some significant differences remained for the genotypes RGM and 161-49C.

Plants were irrigated at field capacity and plastic bags were tied around the cutting wood in order to prevent water loss from substrate evaporation. A water-deficit treatment was applied by withholding irrigation for 4 days. Plants were sampled daily from day 1 (24 h after the last irrigation, defined as non-stressed) to day 4 (water-stressed), during the last hour of night period. Three plants per genotype were used for water potential measurements and xylem sap sampling, and three plants were sampled for gene expression analysis (all of the 2 cm long root tips and all leaves (*n* = 7–10)). Just before sampling, leaf area of each plant was determined for the six plants as described above. Fresh biomass was determined for each compartment (leaves, stem, cutting and roots) for all samples. All pots were weighed daily during the last hours of the night, prior to sampling, to calculate daily transpiration.

### Determination of water potential and xylem sap collection

Each plant stem was first cut at 5 cm above its basal end. The basal part, including the roots, cutting and some stem, was considered as the root part. Then the upper part of the stem was cut at 2 cm under the fifth apical leaf and the apical section was considered as the shoot part. The root (still enclosed in the plastic bag) and shoot parts were inserted concomitantly into two pressure chambers equipped with digital LCD manometers (SAM Précis 2000, Gradignan, France) to measure simultaneously root and stem water potential. When equilibrium of pressure was obtained and water potential recorded, an over-pressure of 0.5 MPa was used for xylem sap collection (approximately 35 μL) after removing of the first drop of xylem sap. Xylem sap samples were immediately frozen in liquid nitrogen and stored at -80 °C prior to freeze-drying (Alpha LSC 1-4, Christ, Germany) and subsequent analysis.

### Analysis of ABA and its derivatives

[ABA], [PA] and [DPA] in xylem sap were measured using liquid chromatography/mass spectrometry (Agilent 6410 Triple Quadrupole LC-MS/MS with Agilent 1200 series HPLC, Agilent Technologies Inc., Santa Clara, USA) using a stable isotope dilution assay [30]. The dry samples of xylem sap were dissolved in 30 μL 10 % acetonitrile (v/v) containing 0.05 % acetic acid (v/v). This acetonitrile solution also contained the deuterated internal standards D3-7′,7′,7′-DPA, D3-7′,7′,7′-PA and D6-3′,5′,5′,7′,7′,7′-ABA, all at a concentration of 100 pg/μL. The column used was a Phenomenex C18(2) 75 mm × 4.5 mm × 5 μm and column temperature was set at 40 °C. The solvents used were nanopure water and acetonitrile, both added with 0.05 % acetic acid (v/v). Samples were eluted with a linear 15 min gradient starting at 10 % acetonitrile (v/v) and ending with 90 % acetonitrile (v/v). Compounds were identified by retention times (DPA = 7.25–7.75, PA = 9.0–9.5 and ABA = 10.5–11.0 min) and multiple reaction monitoring of mass-to-charge ratio (m/z) for parent and product ions of native (DPA = 281/284, PA = 279/282 and ABA = 263/269) and deuterated internal standards (DPA = 171/174, PA = 139/142 and ABA = 153/159) [30].

### RNA extraction and qPCR

Root tips and entire leaves were snap frozen in liquid nitrogen and ground with a ball mill (MM 400, Retsch GmbH, Hann, Germany). Total RNA was extracted from 150 mg of fresh matter according to Reid et al. [65]. Genomic DNA contamination was removed with the Turbo DNA-free kit (Life technologies, according to the manufacturer’s instructions) and reverse transcription was performed using Superscript III (Invitrogen) using oligo dT primers and 1.5 μg of RNA according to the manufacturer’s instructions. Transcript abundance of *VviNCED1*, *VviNCED2, VviHyd1, VviHyd2, VviRCAR5, VviRCAR6, VviPP2C4, VviPP2C9, VviSnRK2.1, VviSnRK2.6, VviABF1* and *VviABF2* was analysed on a Biorad CFX96 machine using iQ Sybr Green Supermix (according to the manufacturer’s instructions) (Additional file 10). The transcript abundance of studied genes was normalized to geometric mean of *VviGAPDH*, *VviEF1γ* and *VviActin* expression [65]. Their suitability to be used as reference genes on non *V. vinifera* genotypes was tested on leaves and roots. The relative gene transcript abundance was calculated according to the 2^-∆∆C^_T_ method [66]. *VviRCARs*, *VviPP2Cs* and *VviSnRK2s* qPCR primers used were from Boneh et al. [38, 39] and the others were designed using Beacon Designer (version 7, CA, USA) (Additional file 10). PCR efficiency for each primer pair was calculated using LinRegPCR [67].

### Statistical analyses

Treatment effect on shoot water potential was analysed using a Kruskall Wallis test (*p* < 0.05). Genotype effect on biomass allocation and transpiration on day 1 and 4 was determined using a one-way analysis of variance (ANOVA, *p* < 0.05, with Tukey’s Honest Significant Difference (HSD) test). Tissue and genotype effects on transcript abundance in non-stressed and water-stressed plants were determined using a two-way ANOVA (*p* < 0.05, with Tukey’s HSD test). All regressions were fitted using Sigma Plot (Version 11, Systat Software) and, when necessary. Genotype-specific and global (including all genotypes) regressions were established between xylem sap hormone content and water potential in shoot and root. Genotype-specific regressions and global regressions were compared by the procedure defined by Snedecor and Cochran [68] using a Fischer-Snedecor test (*p* < 0.05). The heatmaps for transcript abundance were created using R v.2.15.3 (R Development Core Team, 2008). Discriminant and principal component analyses and the Pearson correlation matrix were done using XLStat (Addinsoft SARL., Paris, France). Principal component analysis was performed on Pearson correlations of raw data. Mean transcript abundance value and ABA metabolite content for each day and for each genotype were used for principal component analysis.

### Ethics

Not applicable.

### Consent to publish

Not applicable.

### Availability of data and materials

Raw data could be obtained by request to the corresponding author.

## Additional files

Additional file 1: Relation between soil water content and root (black circle) and shoot (white circle) water potential (*n* = 101). (TIF 374 kb) Additional file 2: Relation between abscisic acid (ABA) (A), phaseic acid (PA) (B) and dihydrophaseic (DPA) (C) content in root and shoot xylem sap. p-values and R^2^ are presented for each regression (*n* = 101). Linear equation: [ABA]_shoot_ = 0.717 [ABA]_root_ -60.2; [PA]_shoot_ = 0.373 [PA]_root_ -9.6; [DPA]_shoot_ = 0.829 [DPA]_root_ + 11.2. (TIF 977 kb) Additional file 3: Relation between phaseic acid (PA) (A & C) or dihydrophaseic (DPA) (B & D) with abscisic acid (ABA) content in shoot (A & B) and in root (C & D) xylem sap. p-values and R^2^ are presented for each regression (*n* = 101). Linear equation: [PA]_shoot_ = 0.2 [ABA]_shoot_ + 301; [DPA]_shoot_ = 0.026 [ABA]_Shoot_ +5.5; [PA]_root_ = 0.121 [ABA]_root_ +81.8; [DPA]_root_ = 0.036 [ABA]_root_ +9.1. (TIF 467 kb) Additional file 4: The transcript abundance of 12 ABA-related genes in the roots and leaves of nine grapevine genotypes on day 1. p-values from a two-way ANOVA (*n* = 3) are presented in first block of the table for each gene. Genotypes, tissues and interaction effects are presented within the following three bold blocks, values with the same letter are not statistical different (Tukey-HSD). (DOCX 28 kb) Additional file 5: The transcript abundance of 12 ABA-related genes in the leaves of nine grapevine genotypes for non-stressed (day 1) and water-stressed (day 4) plants. p-values from a two-way ANOVA (*n* = 3) are presented in first block of the table for each gene. Genotype, day of sampling and interaction effects are presented within the following three bold blocks, values with the same letter are not statistical different (Tukey-HSD). (DOCX 27 kb) Additional file 6: The transcript abundance of 12 ABA-related genes in the roots of nine grapevine genotypes for non-stressed (day 1) and water-stressed (day 4) plants. p-values from a two-way ANOVA (*n* = 3) are presented in first block of the table for each gene. Genotype, day of sampling and interaction effects are presented within the following three bold blocks, values with the same letter are not statistical different (Tukey-HSD). (DOCX 27 kb) Additional file 7: Pearson correlation matrix of all mean data (*n* = 27). High correlation values are shown in bold (correlation coefficient > 0.7). Transcript abundance are presented in leaves (-L) and in roots (-R). Abbreviations: SWCmeta: soil water content (SWC) for plants coming from water potential measurement; SWCtrans: SWC for plants coming for transcriptional studies; SWP: stem water potential; RWP: root water potential. (DOCX 35 kb) Additional file 8: Coordinates of variables from the discriminant analysis of gene expression data (F1, F2, Fig. 6) and from principle component analysis of mean transcript abundance and metabolite data (PC1, PC2, Fig. 7). Abbreviation: abscisic acid (ABA), phaseic acid (PA) and dihydrophaseic (DPA) content in shoot (-S) and root (-R) sap; transcript abundance in leaves (-L) and roots (-R). (DOCX 12 kb) Additional file 9: Fresh biomass, leaf area and soil water content for nine grapevine genotypes. Values represent mean ± standard deviation (*n* = 24, except for soil water content on day 1 and 4, *n* = 6). Values among genotypes with the same letter are not statistical different (one way ANOVA, p-value is presented in bottom line). (DOCX 20 kb) Additional file 10: Primer pair sequences used in this study. Accessions number from Gramene: http://www.gramene.org. (DOCX 14 kb)

## Abbreviations

ABA: abscisic acid
ABA-GE: ABA-glycosyl ester
ABFs or AREBs: ABA-responsive element binding factors
DPA: dihydrophaseic acid
Hyd: ABA 8′-hydroxylase
NCED: nine-cis-epoxycarotenoid dioxygenase
OST1: open stomata 1
PA: phaseic acid
PP2C: protein phospatase 2C
PYR/PYL/RCAR: pyrabactin resistance 1/PYR1-Like/Regulatory components of ABA receptors
SnRK2: sucrose non-fermenting-related kinase 2
TF: transcription factors
VPD: vapour pressure deficit
