# Supplementary material for: ABA-mediated responses to water deficit separate grapevine genotypes by their genetic background
Source: BMC Plant Biol. 2016 Apr 18;16:91. doi: 10.1186/s12870-016-0778-4 (PMC4836075; doi:10.1186/s12870-016-0778-4)
Supplement: Additional file 4: — The transcript abundance of 12 ABA-related genes in the roots and leaves of nine grapevine genotypes on day 1. p-values from a two-way ANOVA (n = 3) are presented in first block of the table for each gene. Genotypes, tissues and interaction effects are presented within the following three bold blocks, values with the same letter are not statistical different (Tukey-HSD). (DOCX 28 kb) [file 12870_2016_778_MOESM4_ESM.docx]

|  | | NCED1 | | NCED2 | | ABF1 | | ABF2 | | Hyd1 | | Hyd2 | | RCAR5 | | RCAR6 | | SnRK2.1 | | SnRK2.6 | | PP2C4 | | PP2C9 | |
| --- | --- | --- | --- | --- | --- | --- | --- | --- | --- | --- | --- | --- | --- | --- | --- | --- | --- | --- | --- | --- | --- | --- | --- | --- | --- |
| Genotypes | | 0.441 | | 0.001 | | 0.000 | | < 0.0001 | | 0.001 | | 0.010 | | 0.000 | | 0.003 | | < 0.0001 | | < 0.0001 | | 0.017 | | 0.016 | |
| Tissues | | 0.017 | | < 0.0001 | | 0.611 | | 0.025 | | < 0.0001 | | < 0.0001 | | < 0.0001 | | < 0.0001 | | < 0.0001 | | < 0.0001 | | < 0.0001 | | 0.048 | |
| Interaction | | 0.108 | | 0.001 | | 0.130 | | < 0.0001 | | 0.000 | | 0.894 | | 0.339 | | 0.008 | | 0.000 | | 0.001 | | 0.369 | | 0.016 | |
| Genotypes effect | RGM | 0.014 | a | 0.218 | bc | 0.165 | c | 0.702 | ab | 0.245 | bc | 0.325 | ab | 0.201 | bc | 0.049 | b | 0.149 | d | 0.265 | bcd | 0.130 | b | 0.009 | b |
|  | 101-14 Mgt | 0.012 | a | 0.278 | abc | 0.214 | bc | 0.299 | c | 0.408 | abc | 0.328 | ab | 0.177 | c | 0.042 | b | 0.213 | bcd | 0.240 | bccde | 0.158 | ab | 0.004 | b |
|  | SO4 | 0.013 | a | 0.367 | ab | 0.231 | bc | 0.366 | bc | 0.115 | c | 0.651 | a | 0.218 | bc | 0.114 | ab | 0.344 | ab | 0.271 | bc | 0.162 | ab | 0.009 | ab |
|  | 161-49 C | 0.009 | a | 0.391 | a | 0.195 | bc | 0.413 | bc | 0.357 | bc | 0.397 | ab | 0.178 | c | 0.117 | ab | 0.274 | bcd | 0.269 | bc | 0.164 | ab | 0.010 | ab |
|  | 41B Mgt | 0.011 | a | 0.299 | abc | 0.186 | c | 1.039 | a | 0.143 | bc | 0.486 | ab | 0.468 | a | 0.176 | a | 0.311 | abc | 0.165 | cde | 0.095 | b | 0.030 | a |
|  | 110R | 0.027 | a | 0.194 | c | 0.350 | ab | 0.081 | c | 0.255 | bc | 0.258 | b | 0.226 | bc | 0.056 | b | 0.185 | cd | 0.316 | ab | 0.207 | ab | 0.009 | b |
|  | 140Ru | 0.020 | a | 0.314 | abc | 0.415 | a | 0.078 | c | 0.605 | ab | 0.508 | ab | 0.164 | c | 0.027 | b | 0.420 | a | 0.403 | a | 0.247 | a | 0.015 | ab |
|  | Syrah | 0.032 | a | 0.215 | c | 0.237 | bc | 0.698 | b | 0.308 | bc | 0.284 | ab | 0.384 | ab | 0.134 | ab | 0.285 | abcd | 0.139 | d | 0.131 | b | 0.003 | b |
|  | Grenache | 0.013 | a | 0.192 | c | 0.163 | c | 0.404 | bc | 0.875 | a | 0.139 | b | 0.198 | bc | 0.071 | ab | 0.207 | bcd | 0.140 | de | 0.166 | ab | 0.003 | b |
| Tissues effects | Leaves | 0.010 | b | 0.377 | a | 0.232 | a | 0.392 | b | 0.725 | a | 0.237 | b | 0.135 | b | 0.016 | b | 0.318 | a | 0.295 | a | 0.208 | a | 0.007 | b |
|  | Roots | 0.023 | a | 0.171 | bc | 0.247 | a | 0.514 | a | 0.011 | bc | 0.512 | a | 0.357 | a | 0.159 | a | 0.212 | b | 0.196 | b | 0.117 | b | 0.014 | a |
|  |  |  | |  | |  | |  | |  | |  | |  | |  | |  | |  | |  | |  | |
| Interaction | Genotypes | NCED1 | | NCED2 | | ABF1 | | ABF2 | | Hyd1 | | Hyd2 | | RCAR5 | | RCAR6 | | SnRK2.1 | | SnRK2.6 | | PP2C4 | | PP2C9 | |
| Leaves | RGM | 0.010 | a | 0.208 | cde | 0.181 | bcd | 0.747 | bc | 0.490 | bc | 0.162 | a | 0.106 | c | 0.022 | de | 0.157 | cd | 0.341 | abcd | 0.157 | abcd | 0.005 | b |
|  | 101-14 Mgt | 0.010 | a | 0.277 | cde | 0.184 | abcd | 0.306 | bcd | 0.816 | bc | 0.126 | a | 0.082 | c | 0.015 | e | 0.303 | bcd | 0.306 | ancdef | 0.174 | abcd | 0.002 | b |
|  | SO4 | 0.021 | a | 0.560 | ab | 0.264 | abcd | 0.422 | bcd | 0.231 | c | 0.612 | a | 0.071 | c | 0.015 | e | 0.416 | ab | 0.332 | abcde | 0.215 | abcd | 0.012 | b |
|  | 161-49 C | 0.009 | a | 0.604 | a | 0.244 | abcd | 0.400 | bcd | 0.714 | bc | 0.218 | a | 0.131 | c | 0.040 | cde | 0.355 | bc | 0.397 | ab | 0.241 | abcd | 0.010 | b |
|  | 41B Mgt | 0.003 | a | 0.437 | abc | 0.123 | d | 0.372 | bcd | 0.190 | c | 0.364 | a | 0.377 | abc | 0.020 | de | 0.267 | bcd | 0.305 | abcdef | 0.108 | abcd | 0.006 | b |
|  | 110R | 0.005 | a | 0.235 | cde | 0.266 | abcd | 0.058 | d | 0.509 | bc | 0.106 | a | 0.097 | c | 0.005 | e | 0.157 | cd | 0.300 | abcdef | 0.255 | abc | 0.004 | b |
|  | 140Ru | 0.014 | a | 0.425 | abc | 0.384 | abc | 0.083 | d | 1.207 | ab | 0.336 | a | 0.079 | c | 0.006 | e | 0.615 | a | 0.357 | abc | 0.288 | a | 0.014 | b |
|  | Syrah | 0.008 | a | 0.323 | bcd | 0.226 | abcd | 0.586 | bcd | 0.616 | bc | 0.117 | a | 0.158 | c | 0.011 | e | 0.291 | bcd | 0.165 | cdefg | 0.165 | abcd | 0.004 | b |
|  | Grenache | 0.014 | a | 0.327 | bcd | 0.217 | abcd | 0.557 | bcd | 1.749 | a | 0.097 | a | 0.117 | c | 0.008 | e | 0.306 | bcd | 0.150 | defg | 0.268 | ab | 0.005 | b |
| Roots | RGM | 0.018 | a | 0.228 | cde | 0.149 | cd | 0.657 | bc | 0.001 | c | 0.487 | a | 0.295 | abc | 0.076 | cde | 0.140 | cd | 0.189 | cdefg | 0.103 | bcd | 0.013 | b |
|  | 101-14 Mgt | 0.015 | a | 0.278 | cde | 0.244 | abcd | 0.292 | bcd | 0.000 | c | 0.530 | a | 0.272 | abc | 0.068 | cde | 0.123 | cd | 0.174 | cdefg | 0.143 | abcd | 0.006 | b |
|  | SO4 | 0.005 | a | 0.174 | de | 0.198 | abcd | 0.309 | bcd | 0.000 | c | 0.690 | a | 0.365 | abc | 0.212 | abc | 0.271 | bcd | 0.211 | bcdefg | 0.108 | abcd | 0.007 | b |
|  | 161-49 C | 0.009 | a | 0.178 | de | 0.146 | cd | 0.425 | bcd | 0.000 | c | 0.576 | a | 0.224 | c | 0.195 | abcd | 0.193 | bcd | 0.141 | defg | 0.086 | bcd | 0.010 | b |
|  | 41B Mgt | 0.018 | a | 0.161 | de | 0.248 | abcd | 1.707 | a | 0.097 | c | 0.609 | a | 0.559 | ab | 0.332 | a | 0.356 | bc | 0.024 | g | 0.081 | cd | 0.055 | a |
|  | 110R | 0.050 | a | 0.153 | de | 0.435 | ab | 0.104 | d | 0.001 | c | 0.410 | a | 0.355 | abc | 0.107 | bcde | 0.213 | bcd | 0.332 | abcde | 0.159 | abcd | 0.013 | b |
|  | 140Ru | 0.026 | a | 0.203 | cde | 0.446 | a | 0.073 | d | 0.003 | c | 0.679 | a | 0.249 | bc | 0.047 | cde | 0.226 | bcd | 0.448 | a | 0.207 | abcd | 0.016 | b |
|  | Syrah | 0.057 | a | 0.108 | de | 0.247 | abcd | 0.811 | b | 0.000 | c | 0.450 | a | 0.610 | a | 0.257 | ab | 0.280 | bcd | 0.114 | fg | 0.096 | bcd | 0.003 | b |
|  | Grenache | 0.013 | a | 0.056 | e | 0.108 | d | 0.252 | cd | 0.001 | c | 0.181 | a | 0.280 | abc | 0.135 | bcde | 0.109 | d | 0.131 | efg | 0.064 | d | 0.001 | b |
