# Supplementary material for: ABA-mediated responses to water deficit separate grapevine genotypes by their genetic background
Source: BMC Plant Biol. 2016 Apr 18;16:91. doi: 10.1186/s12870-016-0778-4 (PMC4836075; doi:10.1186/s12870-016-0778-4)
Supplement: Additional file 5: — The transcript abundance of 12 ABA-related genes in the leaves of nine grapevine genotypes for non-stressed (day 1) and water-stressed (day 4) plants. p-values from a two-way ANOVA (n = 3) are presented in first block of the table for each gene. Genotype, day of sampling and interaction effects are presented within the following three bold blocks, values with the same letter are not statistical different (Tukey-HSD). (DOCX 27 kb) [file 12870_2016_778_MOESM5_ESM.docx]

|  |  | NCED1 | | NCED2 | | ABF1 | | ABF2 | | Hyd1 | | Hyd2 | | RCAR5 | | RCAR6 | | SnRK2.1 | | SnRK2.6 | | PP2C4 | | PP2C9 | |
| --- | --- | --- | --- | --- | --- | --- | --- | --- | --- | --- | --- | --- | --- | --- | --- | --- | --- | --- | --- | --- | --- | --- | --- | --- | --- |
| Genotypes | | 0.000 | | < 0.0001 | | < 0.0001 | | < 0.0001 | | < 0.0001 | | 0.002 | | < 0.0001 | | < 0.0001 | | < 0.0001 | | < 0.0001 | | 0.002 | | 0.001 | |
| Day | | < 0.0001 | | 0.461 | | < 0.0001 | | < 0.0001 | | 0.014 | | < 0.0001 | | < 0.0001 | | < 0.0001 | | < 0.0001 | | 0.000 | | < 0.0001 | | < 0.0001 | |
| Interaction | | 0.000 | | 0.002 | | < 0.0001 | | < 0.0001 | | 0.002 | | 0.000 | | < 0.0001 | | 0.074 | | 0.113 | | 0.002 | | 0.001 | | 0.002 | |
| Genotypes effect | RGM | 0.238 | b | 0.343 | cd | 0.394 | cd | 1.436 | a | 0.670 | bc | 0.511 | ab | 0.059 | b | 0.013 | b | 0.278 | d | 0.413 | a | 0.515 | bc | 0.027 | b |
|  | 101-14 Mgt | 0.210 | b | 0.344 | cd | 0.491 | cd | 0.438 | de | 0.644 | bc | 0.190 | b | 0.044 | b | 0.009 | b | 0.377 | bcd | 0.308 | b | 0.423 | c | 0.018 | b |
|  | SO4 | 0.046 | b | 0.537 | ab | 0.307 | d | 0.536 | d | 0.284 | bc | 0.619 | ab | 0.044 | b | 0.011 | b | 0.592 | abc | 0.315 | b | 0.375 | c | 0.023 | b |
|  | 161-49 C | 0.234 | b | 0.612 | a | 0.586 | bcd | 0.640 | cd | 0.671 | bc | 0.526 | ab | 0.079 | b | 0.027 | a | 0.635 | ab | 0.431 | a | 0.599 | abc | 0.097 | ab |
|  | 41B Mgt | 0.383 | ab | 0.351 | cd | 0.704 | bcd | 0.883 | bc | 0.171 | c | 0.495 | ab | 0.192 | a | 0.011 | b | 0.331 | cd | 0.370 | ab | 1.110 | ab | 0.077 | ab |
|  | 110R | 0.735 | ab | 0.311 | cd | 0.928 | bcd | 0.114 | e | 0.535 | bc | 1.034 | a | 0.051 | b | 0.003 | b | 0.252 | d | 0.320 | b | 0.761 | abc | 0.048 | ab |
|  | 140Ru | 0.613 | ab | 0.430 | bc | 1.056 | abc | 0.149 | e | 0.734 | b | 0.981 | a | 0.041 | b | 0.004 | b | 0.838 | a | 0.341 | b | 0.629 | abc | 0.131 | a |
|  | Syrah | 1.070 | a | 0.288 | cd | 1.628 | a | 1.160 | ab | 0.635 | bc | 0.527 | ab | 0.080 | b | 0.006 | b | 0.329 | cd | 0.186 | c | 0.948 | abc | 0.094 | ab |
|  | Grenache | 1.079 | a | 0.272 | d | 1.246 | ab | 1.352 | a | 1.240 | a | 0.664 | ab | 0.060 | b | 0.004 | b | 0.565 | abc | 0.162 | c | 1.187 | a | 0.142 | a |
| Day effects | Day 1 | 0.010 | b | 0.377 | a | 0.232 | b | 0.392 | b | 0.725 | a | 0.237 | b | 0.135 | a | 0.016 | a | 0.318 | b | 0.295 | b | 0.208 | b | 0.007 | b |
|  | Day 4 | 1.013 | a | 0.397 | a | 1.399 | a | 1.098 | a | 0.516 | b | 0.995 | a | 0.009 | b | 0.003 | b | 0.614 | a | 0.338 | a | 1.247 | a | 0.139 | a |
|  |  |  | |  | |  | |  | |  | |  | |  | |  | |  | |  | |  | |  | |
| Interaction | Category | NCED1 | | NCED2 | | ABF1 | | ABF2 | | Hyd1 | | Hyd2 | | RCAR5 | | RCAR6 | | SnRK2.1 | | SnRK2.6 | | PP2C4 | | PP2C9 | |
| Day 1 | RGM | 0.010 | d | 0.208 | e | 0.181 | f | 0.747 | de | 0.490 | bc | 0.162 | d | 0.106 | bcd | 0.022 | b | 0.157 | d | 0.341 | bc | 0.157 | d | 0.005 | c |
|  | 101-14 Mgt | 0.010 | d | 0.277 | cde | 0.184 | ef | 0.306 | defg | 0.816 | bc | 0.126 | d | 0.082 | bcdef | 0.015 | bcd | 0.303 | d | 0.306 | cd | 0.174 | d | 0.002 | c |
|  | SO4 | 0.021 | d | 0.560 | ab | 0.264 | ef | 0.422 | defg | 0.231 | c | 0.612 | cd | 0.071 | bcdef | 0.015 | bcd | 0.416 | bcd | 0.332 | bc | 0.215 | d | 0.012 | c |
|  | 161-49 C | 0.009 | d | 0.604 | a | 0.244 | ef | 0.400 | defg | 0.714 | bc | 0.218 | d | 0.131 | b | 0.040 | a | 0.355 | cd | 0.397 | abc | 0.241 | cd | 0.010 | c |
|  | 41B Mgt | 0.003 | d | 0.437 | abcde | 0.123 | f | 0.372 | defg | 0.190 | c | 0.364 | cd | 0.377 | a | 0.020 | bc | 0.267 | d | 0.305 | cd | 0.108 | d | 0.006 | c |
|  | 110R | 0.005 | d | 0.235 | e | 0.266 | ef | 0.058 | g | 0.509 | bc | 0.106 | d | 0.097 | bcde | 0.005 | bcd | 0.157 | d | 0.300 | cd | 0.255 | cd | 0.004 | c |
|  | 140Ru | 0.014 | d | 0.425 | abcde | 0.384 | ef | 0.083 | g | 1.207 | ab | 0.336 | cd | 0.079 | bcdef | 0.006 | bcd | 0.615 | abcd | 0.357 | bc | 0.288 | cd | 0.014 | c |
|  | Syrah | 0.008 | d | 0.323 | bcde | 0.226 | ef | 0.586 | defg | 0.616 | bc | 0.117 | d | 0.158 | b | 0.011 | bcd | 0.291 | d | 0.165 | e | 0.165 | d | 0.004 | c |
|  | Grenache | 0.014 | d | 0.327 | bcde | 0.217 | ef | 0.557 | defg | 1.749 | a | 0.097 | d | 0.117 | bc | 0.008 | bcd | 0.306 | d | 0.150 | e | 0.268 | cd | 0.005 | c |
| Day 4 | RGM | 0.465 | bcd | 0.477 | abcd | 0.607 | def | 2.125 | a | 0.849 | bc | 0.860 | bcd | 0.013 | ef | 0.004 | cd | 0.399 | bcd | 0.485 | a | 0.873 | bcd | 0.049 | bc |
|  | 101-14 Mgt | 0.410 | bcd | 0.410 | abcde | 0.798 | cdef | 0.570 | defg | 0.471 | bc | 0.254 | d | 0.005 | ef | 0.002 | d | 0.451 | bcd | 0.310 | cd | 0.672 | cd | 0.035 | bc |
|  | SO4 | 0.072 | cd | 0.513 | abc | 0.350 | ef | 0.650 | def | 0.337 | c | 0.626 | cd | 0.016 | def | 0.006 | bcd | 0.769 | abc | 0.298 | cd | 0.534 | cd | 0.035 | bc |
|  | 161-49 C | 0.459 | bcd | 0.620 | a | 0.929 | cdef | 0.879 | cd | 0.628 | bc | 0.835 | bcd | 0.026 | cdef | 0.014 | bcd | 0.914 | a | 0.465 | a | 0.958 | bcd | 0.183 | ab |
|  | 41B Mgt | 0.763 | bcd | 0.266 | de | 1.285 | bcde | 1.394 | bc | 0.151 | c | 0.627 | cd | 0.007 | ef | 0.001 | d | 0.395 | bcd | 0.435 | ab | 2.112 | a | 0.149 | abc |
|  | 110R | 1.464 | ab | 0.387 | abcde | 1.591 | bcd | 0.169 | fg | 0.561 | bc | 1.962 | a | 0.004 | f | 0.001 | d | 0.347 | cd | 0.341 | bc | 1.267 | abc | 0.093 | bc |
|  | 140Ru | 1.212 | abc | 0.435 | abcde | 1.728 | bc | 0.215 | efg | 0.262 | c | 1.625 | ab | 0.002 | f | 0.002 | d | 1.062 | a | 0.325 | c | 0.970 | bcd | 0.248 | a |
|  | Syrah | 2.133 | a | 0.252 | de | 3.031 | a | 1.735 | ab | 0.654 | bc | 0.937 | bcd | 0.002 | f | 0.000 | d | 0.367 | bcd | 0.207 | de | 1.731 | ab | 0.183 | ab |
|  | Grenache | 2.144 | a | 0.216 | e | 2.275 | ab | 2.147 | a | 0.730 | bc | 1.231 | abc | 0.003 | f | 0.001 | d | 0.825 | ab | 0.174 | e | 2.107 | a | 0.278 | a |
