# Supplementary material for: ABA-mediated responses to water deficit separate grapevine genotypes by their genetic background
Source: BMC Plant Biol. 2016 Apr 18;16:91. doi: 10.1186/s12870-016-0778-4 (PMC4836075; doi:10.1186/s12870-016-0778-4)
Supplement: Additional file 6: — The transcript abundance of 12 ABA-related genes in the roots of nine grapevine genotypes for non-stressed (day 1) and water-stressed (day 4) plants. p-values from a two-way ANOVA (n = 3) are presented in first block of the table for each gene. Genotype, day of sampling and interaction effects are presented within the following three bold blocks, values with the same letter are not statistical different (Tukey-HSD). (DOCX 27 kb) [file 12870_2016_778_MOESM6_ESM.docx]

|  | | NCED1 | | NCED2 | | ABF1 | | ABF2 | | Hyd1 | | Hyd2 | | RCAR5 | | RCAR6 | | SnRK2.1 | | SnRK2.6 | | PP2C4 | | PP2C9 | |
| --- | --- | --- | --- | --- | --- | --- | --- | --- | --- | --- | --- | --- | --- | --- | --- | --- | --- | --- | --- | --- | --- | --- | --- | --- | --- |
| Genotypes | | 0.069 | | 0.000 | | 0.065 | | 0.001 | | 0.000 | | 0.088 | | 0.080 | | 0.015 | | 0.248 | | < 0.0001 | | 0.465 | | 0.012 | |
| Day | | < 0.0001 | | < 0.0001 | | < 0.0001 | | 0.001 | | 0.294 | | < 0.0001 | | < 0.0001 | | < 0.0001 | | < 0.0001 | | 0.138 | | < 0.0001 | | < 0.0001 | |
| Interaction | | 0.135 | | 0.010 | | 0.822 | | 0.543 | | 0.001 | | 0.443 | | 0.031 | | 0.019 | | 0.597 | | 0.212 | | 0.339 | | 0.225 | |
| Genotype effect | RGM | 0.246 | a | 0.701 | b | 0.381 | a | 0.908 | ab | 0.001 | b | 1.234 | a | 0.190 | a | 0.057 | ab | 0.186 | a | 0.141 | bc | 0.240 | a | 0.037 | ab |
|  | 101-14 Mgt | 0.761 | a | 1.363 | a | 0.613 | a | 0.866 | ab | 0.001 | b | 0.701 | ab | 0.190 | a | 0.047 | ab | 0.370 | a | 0.117 | bc | 0.326 | a | 0.027 | ab |
|  | SO4 | 0.068 | a | 0.546 | b | 0.356 | a | 0.594 | b | 0.000 | b | 1.047 | ab | 0.220 | a | 0.170 | a | 0.324 | a | 0.179 | bc | 0.267 | a | 0.056 | ab |
|  | 161-49 C | 0.335 | a | 0.549 | b | 0.515 | a | 0.845 | ab | 0.000 | b | 0.604 | ab | 0.142 | a | 0.125 | ab | 0.409 | a | 0.121 | bc | 0.258 | a | 0.061 | ab |
|  | 41B Mgt | 0.176 | a | 0.372 | b | 0.711 | a | 1.773 | a | 0.053 | a | 0.927 | ab | 0.306 | a | 0.168 | a | 0.635 | a | 0.099 | c | 0.490 | a | 0.090 | a |
|  | 110R | 0.501 | a | 0.456 | b | 0.815 | a | 0.176 | b | 0.006 | b | 0.790 | ab | 0.209 | a | 0.062 | ab | 0.505 | a | 0.266 | ab | 0.260 | a | 0.023 | b |
|  | 140Ru | 0.213 | a | 0.589 | b | 0.877 | a | 0.143 | b | 0.016 | b | 1.111 | ab | 0.143 | a | 0.033 | b | 0.514 | a | 0.409 | a | 0.442 | a | 0.081 | ab |
|  | Syrah | 0.791 | a | 0.507 | b | 0.612 | a | 0.960 | ab | 0.001 | b | 0.640 | ab | 0.319 | a | 0.133 | ab | 0.416 | a | 0.115 | bc | 0.483 | a | 0.037 | ab |
|  | Grenache | 0.478 | a | 0.399 | b | 0.374 | a | 0.862 | ab | 0.000 | b | 0.326 | b | 0.150 | a | 0.069 | ab | 0.371 | a | 0.144 | bc | 0.383 | a | 0.023 | b |
| Day effects | Day 1 | 0.023 | b | 0.171 | b | 0.247 | b | 0.514 | b | 0.011 | a | 0.512 | b | 0.357 | a | 0.159 | a | 0.212 | b | 0.196 | a | 0.117 | b | 0.014 | b |
|  | Day 4 | 0.770 | a | 1.047 | a | 0.921 | a | 1.069 | a | 0.006 | a | 1.127 | a | 0.059 | b | 0.033 | b | 0.616 | a | 0.157 | a | 0.583 | a | 0.083 | a |
|  |  |  |  |  |  |  |  |  |  |  |  |  |  |  |  |  |  |  |  |  |  |  |  |  |  |
| Interaction | Category | NCED1 | | NCED2 | | ABF1 | | ABF2 | | Hyd1 | | Hyd2 | | RCAR5 | | RCAR6 | | SnRK2.1 | | SnRK2.6 | | PP2C4 | | PP2C9 | |
| Day 1 | RGM | 0.018 | b | 0.228 | cd | 0.149 | c | 0.657 | abc | 0.001 | b | 0.487 | b | 0.295 | abcde | 0.076 | bc | 0.140 | b | 0.189 | bcd | 0.103 | c | 0.013 | cde |
|  | 101-14 Mgt | 0.015 | b | 0.278 | bcd | 0.244 | bc | 0.292 | abc | 0.000 | b | 0.530 | ab | 0.272 | bcde | 0.068 | bc | 0.123 | b | 0.174 | bcd | 0.143 | bc | 0.006 | de |
|  | SO4 | 0.005 | b | 0.174 | cd | 0.198 | c | 0.309 | abc | 0.000 | b | 0.690 | ab | 0.365 | abc | 0.212 | abc | 0.271 | ab | 0.211 | abcd | 0.108 | c | 0.007 | de |
|  | 161-49 C | 0.009 | b | 0.178 | cd | 0.146 | c | 0.425 | abc | 0.000 | b | 0.576 | ab | 0.224 | cde | 0.195 | abc | 0.193 | ab | 0.141 | bcd | 0.086 | c | 0.010 | de |
|  | 41B Mgt | 0.018 | b | 0.161 | cd | 0.248 | bc | 1.707 | ab | 0.097 | a | 0.609 | ab | 0.559 | ab | 0.332 | a | 0.356 | ab | 0.024 | d | 0.081 | c | 0.055 | abcde |
|  | 110R | 0.050 | b | 0.153 | cd | 0.435 | abc | 0.104 | c | 0.001 | b | 0.410 | b | 0.355 | abcd | 0.107 | bc | 0.213 | ab | 0.332 | abc | 0.159 | bc | 0.013 | cde |
|  | 140Ru | 0.026 | b | 0.203 | cd | 0.446 | abc | 0.073 | c | 0.003 | b | 0.679 | ab | 0.249 | bcde | 0.047 | bc | 0.226 | ab | 0.448 | a | 0.207 | abc | 0.016 | cde |
|  | Syrah | 0.057 | b | 0.108 | cd | 0.247 | bc | 0.811 | abc | 0.000 | b | 0.450 | b | 0.610 | a | 0.257 | ab | 0.280 | ab | 0.114 | cd | 0.096 | c | 0.003 | e |
|  | Grenache | 0.013 | b | 0.056 | d | 0.108 | c | 0.252 | bc | 0.001 | b | 0.181 | b | 0.280 | bcde | 0.135 | abc | 0.109 | b | 0.131 | bcd | 0.064 | c | 0.001 | e |
| Day 4 | RGM | 0.473 | ab | 1.175 | b | 0.613 | abc | 1.158 | abc | 0.001 | b | 1.981 | a | 0.086 | cde | 0.038 | c | 0.232 | ab | 0.092 | cd | 0.377 | abc | 0.061 | abcde |
|  | 101-14 Mgt | 1.507 | a | 2.448 | a | 0.981 | abc | 1.440 | abc | 0.001 | b | 0.871 | ab | 0.107 | cde | 0.026 | c | 0.618 | ab | 0.061 | d | 0.510 | abc | 0.049 | abcde |
|  | SO4 | 0.131 | b | 0.918 | bcd | 0.515 | abc | 0.878 | abc | 0.000 | b | 1.403 | ab | 0.074 | cde | 0.127 | abc | 0.376 | ab | 0.147 | bcd | 0.426 | abc | 0.105 | abcd |
|  | 161-49 C | 0.661 | ab | 0.920 | bcd | 0.884 | abc | 1.265 | abc | 0.001 | b | 0.631 | ab | 0.060 | cde | 0.056 | bc | 0.624 | ab | 0.100 | cd | 0.429 | abc | 0.112 | abc |
|  | 41B Mgt | 0.334 | ab | 0.584 | bcd | 1.173 | ab | 1.838 | a | 0.010 | b | 1.245 | ab | 0.054 | cde | 0.004 | c | 0.915 | a | 0.174 | bcd | 0.899 | a | 0.124 | ab |
|  | 110R | 0.952 | ab | 0.759 | bcd | 1.194 | a | 0.248 | bc | 0.011 | b | 1.169 | ab | 0.063 | cde | 0.018 | c | 0.797 | ab | 0.199 | abcd | 0.360 | abc | 0.034 | bcde |
|  | 140Ru | 0.400 | ab | 0.975 | bc | 1.309 | a | 0.212 | bc | 0.029 | b | 1.544 | ab | 0.036 | de | 0.018 | c | 0.801 | ab | 0.370 | ab | 0.678 | abc | 0.145 | a |
|  | Syrah | 1.525 | a | 0.907 | bcd | 0.977 | abc | 1.110 | abc | 0.001 | b | 0.830 | ab | 0.028 | e | 0.008 | c | 0.552 | ab | 0.116 | cd | 0.870 | ab | 0.071 | abcde |
|  | Grenache | 0.943 | ab | 0.742 | bcd | 0.640 | abc | 1.472 | abc | 0.000 | b | 0.471 | b | 0.020 | e | 0.004 | c | 0.632 | ab | 0.157 | bcd | 0.702 | abc | 0.045 | bcde |
