# Supplementary material for: ABA-mediated responses to water deficit separate grapevine genotypes by their genetic background
Source: BMC Plant Biol. 2016 Apr 18;16:91. doi: 10.1186/s12870-016-0778-4 (PMC4836075; doi:10.1186/s12870-016-0778-4)
Supplement: Additional file 7: — Pearson correlation matrix of all mean data (n = 27). High correlation values are shown in bold (correlation coefficient > 0.7). Transcript abundance are presented in leaves (-L) and in roots (-R). Abbreviations: SWCmeta: soil water content (SWC) for plants coming from water potential measurement; SWCtrans: SWC for plants coming for transcriptional studies; SWP: stem water potential; RWP: root water potential. (DOCX 35 kb) [file 12870_2016_778_MOESM7_ESM.docx]

| **Variables** | **ABA_Stem_** | **PAF_Stem_** | **DPA_Stem_** | **ABA_Root_** | **PA_Root_** | **DPA_Root_** | **NCED1L** | **NCED2L** | **ABF1L** | **ABF2L** | **RCAR5L** | **RCAR6L** | **SnRK2.1L** | **SnRK2.6L** | **PP2C4L** | **PP2C9L** | **NCED1R** |
| --- | --- | --- | --- | --- | --- | --- | --- | --- | --- | --- | --- | --- | --- | --- | --- | --- | --- |
| **ABA_Stem_** | **1.00** | **0.84** | **0.78** | **0.96** | **0.84** | **0.90** | **0.81** | -0.32 | **0.81** | 0.63 | -0.48 | -0.59 | 0.48 | -0.34 | **0.86** | **0.73** | 0.67 |
| **PAF_Stem_** | **0.84** | **1.00** | 0.39 | **0.86** | **0.94** | 0.57 | 0.55 | -0.18 | 0.48 | 0.53 | -0.33 | -0.43 | 0.51 | -0.18 | 0.69 | 0.65 | 0.35 |
| **DPA_Stem_** | **0.78** | 0.39 | **1.00** | **0.73** | 0.41 | **0.91** | **0.81** | -0.47 | **0.87** | 0.62 | -0.32 | -0.48 | 0.18 | -0.45 | **0.76** | 0.60 | 0.64 |
| **ABA_Root_** | **0.96** | **0.86** | **0.73** | **1.00** | **0.92** | **0.85** | **0.83** | -0.30 | **0.81** | 0.66 | -0.47 | -0.57 | 0.57 | -0.38 | **0.82** | **0.80** | 0.66 |
| **PA_Root_** | **0.84** | **0.94** | 0.41 | **0.92** | **1.00** | 0.61 | 0.63 | -0.14 | 0.56 | 0.57 | -0.41 | -0.46 | 0.63 | -0.24 | 0.64 | **0.70** | 0.49 |
| **DPA_Root_** | **0.90** | 0.57 | **0.91** | **0.85** | 0.61 | **1.00** | **0.89** | -0.35 | **0.92** | 0.58 | -0.47 | -0.60 | 0.34 | -0.33 | **0.85** | **0.72** | **0.72** |
| **NCED1L** | **0.81** | 0.55 | **0.81** | **0.83** | 0.63 | **0.89** | **1.00** | -0.33 | **0.97** | 0.50 | -0.49 | -0.55 | 0.36 | -0.29 | **0.86** | **0.83** | **0.73** |
| **NCED2L** | -0.32 | -0.18 | -0.47 | -0.30 | -0.14 | -0.35 | -0.33 | **1.00** | -0.32 | -0.28 | 0.00 | 0.37 | 0.36 | 0.51 | -0.33 | -0.13 | -0.19 |
| **ABF1L** | **0.81** | 0.48 | **0.87** | **0.81** | 0.56 | **0.92** | **0.97** | -0.32 | **1.00** | 0.51 | -0.53 | -0.57 | 0.40 | -0.31 | **0.85** | **0.82** | **0.78** |
| **ABF2L** | 0.63 | 0.53 | 0.62 | 0.66 | 0.57 | 0.58 | 0.50 | -0.28 | 0.51 | **1.00** | -0.31 | -0.29 | 0.16 | -0.11 | 0.63 | 0.43 | 0.51 |
| **RCAR5L** | -0.48 | -0.33 | -0.32 | -0.47 | -0.41 | -0.47 | -0.49 | 0.00 | -0.53 | -0.31 | **1.00** | 0.57 | -0.42 | -0.09 | -0.57 | -0.48 | -0.51 |
| **RCAR6L** | -0.59 | -0.43 | -0.48 | -0.57 | -0.46 | -0.60 | -0.55 | 0.37 | -0.57 | -0.29 | 0.57 | **1.00** | -0.32 | 0.30 | -0.58 | -0.46 | -0.49 |
| **SnRK2.1L** | 0.48 | 0.51 | 0.18 | 0.57 | 0.63 | 0.34 | 0.36 | 0.36 | 0.40 | 0.16 | -0.42 | -0.32 | **1.00** | -0.05 | 0.35 | 0.66 | 0.30 |
| **SnRK2.6L** | -0.34 | -0.18 | -0.45 | -0.38 | -0.24 | -0.33 | -0.29 | 0.51 | -0.31 | -0.11 | -0.09 | 0.30 | -0.05 | **1.00** | -0.07 | -0.10 | -0.28 |
| **PP2C4L** | **0.86** | 0.69 | **0.76** | **0.82** | 0.64 | **0.85** | **0.86** | -0.33 | **0.85** | 0.63 | -0.57 | -0.58 | 0.35 | -0.07 | **1.00** | **0.83** | 0.64 |
| **PP2C9L** | **0.73** | 0.65 | 0.60 | **0.80** | **0.70** | **0.72** | **0.83** | -0.13 | **0.82** | 0.43 | -0.48 | -0.46 | 0.66 | -0.10 | **0.83** | **1.00** | 0.51 |
| **NCED1R** | 0.67 | 0.35 | 0.64 | 0.66 | 0.49 | **0.72** | **0.73** | -0.19 | **0.78** | 0.51 | -0.51 | -0.49 | 0.30 | -0.28 | 0.64 | 0.51 | **1.00** |
| **NCED2R** | 0.19 | 0.08 | 0.07 | 0.22 | 0.21 | 0.20 | 0.30 | 0.14 | 0.32 | 0.19 | -0.51 | -0.31 | 0.26 | 0.20 | 0.35 | 0.27 | 0.66 |
| **ABF1R** | 0.47 | 0.37 | 0.30 | 0.45 | 0.41 | 0.50 | 0.58 | 0.03 | 0.61 | 0.18 | -0.56 | -0.47 | 0.42 | 0.12 | 0.63 | 0.57 | 0.67 |
| **ABF2R** | 0.33 | 0.32 | 0.30 | 0.33 | 0.28 | 0.25 | 0.21 | -0.07 | 0.24 | 0.54 | 0.01 | 0.00 | 0.10 | 0.00 | 0.42 | 0.24 | 0.49 |
| **RCAR5R** | -0.55 | -0.46 | -0.32 | -0.56 | -0.53 | -0.53 | -0.56 | -0.14 | -0.58 | -0.41 | **0.83** | 0.43 | -0.53 | -0.24 | -0.66 | -0.61 | -0.55 |
| **RCAR6R** | -0.56 | -0.42 | -0.37 | -0.54 | -0.48 | -0.53 | -0.54 | 0.22 | -0.57 | -0.38 | **0.85** | 0.60 | -0.37 | -0.14 | -0.63 | -0.53 | -0.55 |
| **SnRK2.1R** | 0.65 | 0.54 | 0.48 | 0.61 | 0.51 | 0.62 | 0.67 | -0.08 | **0.70** | 0.22 | -0.47 | -0.48 | 0.48 | -0.04 | **0.76** | **0.70** | 0.67 |
| **SnRK2.6R** | -0.03 | 0.07 | -0.16 | -0.01 | 0.07 | -0.03 | 0.01 | -0.03 | 0.01 | -0.45 | -0.21 | -0.27 | 0.23 | 0.07 | -0.08 | 0.11 | -0.31 |
| **PP2C4R** | 0.67 | 0.48 | 0.64 | 0.66 | 0.48 | **0.71** | 0.67 | -0.28 | **0.74** | 0.48 | -0.61 | -0.57 | 0.40 | -0.08 | **0.84** | **0.72** | 0.65 |
| **PP2C9R** | 0.32 | 0.30 | 0.21 | 0.34 | 0.30 | 0.37 | 0.36 | 0.21 | 0.40 | 0.15 | -0.32 | -0.31 | 0.61 | 0.32 | 0.53 | 0.66 | 0.23 |
| **Hyd1L** | 0.12 | 0.06 | 0.13 | 0.12 | 0.12 | 0.06 | 0.03 | -0.09 | 0.06 | 0.17 | -0.06 | -0.06 | 0.10 | -0.34 | -0.08 | -0.10 | 0.14 |
| **Hyd1R** | -0.11 | -0.01 | -0.11 | -0.10 | -0.07 | -0.10 | -0.05 | 0.08 | -0.09 | -0.21 | 0.69 | 0.16 | -0.03 | 0.04 | -0.12 | -0.01 | -0.14 |
| **Hyd2L** | 0.57 | 0.51 | 0.38 | 0.58 | 0.56 | 0.59 | **0.73** | 0.08 | 0.66 | 0.17 | -0.44 | -0.45 | 0.51 | 0.09 | 0.62 | **0.73** | 0.41 |
| **Hyd2R** | 0.08 | 0.07 | -0.01 | 0.07 | 0.09 | 0.13 | 0.16 | 0.15 | 0.17 | 0.03 | -0.44 | -0.36 | 0.13 | 0.36 | 0.26 | 0.18 | 0.13 |
| **SWCmeta** | -0.66 | -0.47 | -0.47 | -0.63 | -0.53 | -0.66 | -0.64 | -0.08 | -0.67 | -0.40 | 0.66 | 0.66 | -0.56 | -0.06 | -**0.74** | -0.66 | -0.68 |
| **SWCtrans** | -0.57 | -0.42 | -0.40 | -0.56 | -0.48 | -0.57 | -0.60 | -0.09 | -0.62 | -0.41 | 0.69 | 0.60 | -0.48 | -0.16 | -**0.71** | -0.62 | -0.65 |
| **SWP** | -**0.87** | -**0.71** | -0.63 | -**0.84** | -**0.74** | -**0.81** | -**0.77** | 0.22 | -**0.76** | -0.53 | 0.59 | **0.70** | -0.47 | 0.15 | -**0.83** | -**0.70** | -**0.76** |
| **RWP** | -**0.89** | -**0.73** | -0.65 | -**0.87** | -**0.77** | -**0.84** | -**0.79** | 0.20 | -**0.80** | -0.50 | 0.59 | 0.68 | -0.56 | 0.14 | -**0.85** | -**0.78** | -**0.70** |

| **Variables** | **NCED2R** | **ABF1R** | **ABF2R** | **RCAR5R** | **RCAR6R** | **SnRK2.1R** | **SnRK2.6R** | **PP2C4R** | **PP2C9R** | **Hyd1L** | **Hyd1R** | **Hyd2L** | **Hyd2R** | **SWCmeta** | **SWCtrans** | **SWP** | **RWP** |
| --- | --- | --- | --- | --- | --- | --- | --- | --- | --- | --- | --- | --- | --- | --- | --- | --- | --- |
| **ABA_Stem_** | 0.19 | 0.47 | 0.33 | -0.55 | -0.56 | 0.65 | -0.03 | 0.67 | 0.32 | 0.12 | -0.11 | 0.57 | 0.08 | -0.66 | -0.57 | -**0.87** | -**0.89** |
| **PAF_Stem_** | 0.08 | 0.37 | 0.32 | -0.46 | -0.42 | 0.54 | 0.07 | 0.48 | 0.30 | 0.06 | -0.01 | 0.51 | 0.07 | -0.47 | -0.42 | -**0.71** | -**0.73** |
| **DPA_Stem_** | 0.07 | 0.30 | 0.30 | -0.32 | -0.37 | 0.48 | -0.16 | 0.64 | 0.21 | 0.13 | -0.11 | 0.38 | -0.01 | -0.47 | -0.40 | -0.63 | -0.65 |
| **ABA_Root_** | 0.22 | 0.45 | 0.33 | -0.56 | -0.54 | 0.61 | -0.01 | 0.66 | 0.34 | 0.12 | -0.10 | 0.58 | 0.07 | -0.63 | -0.56 | -**0.84** | -**0.87** |
| **PA_Root_** | 0.21 | 0.41 | 0.28 | -0.53 | -0.48 | 0.51 | 0.07 | 0.48 | 0.30 | 0.12 | -0.07 | 0.56 | 0.09 | -0.53 | -0.48 | -**0.74** | -**0.77** |
| **DPA_Root_** | 0.20 | 0.50 | 0.25 | -0.53 | -0.53 | 0.62 | -0.03 | **0.71** | 0.37 | 0.06 | -0.10 | 0.59 | 0.13 | -0.66 | -0.57 | -**0.81** | -**0.84** |
| **NCED1L** | 0.30 | 0.58 | 0.21 | -0.56 | -0.54 | 0.67 | 0.01 | 0.67 | 0.36 | 0.03 | -0.05 | **0.73** | 0.16 | -0.64 | -0.60 | -**0.77** | -**0.79** |
| **NCED2L** | 0.14 | 0.03 | -0.07 | -0.14 | 0.22 | -0.08 | -0.03 | -0.28 | 0.21 | -0.09 | 0.08 | 0.08 | 0.15 | -0.08 | -0.09 | 0.22 | 0.20 |
| **ABF1L** | 0.32 | 0.61 | 0.24 | -0.58 | -0.57 | **0.70** | 0.01 | 0.74 | 0.40 | 0.06 | -0.09 | 0.66 | 0.17 | -0.67 | -0.62 | -**0.76** | -**0.80** |
| **ABF2L** | 0.19 | 0.18 | 0.54 | -0.41 | -0.38 | 0.22 | -0.45 | 0.48 | 0.15 | 0.17 | -0.21 | 0.17 | 0.03 | -0.40 | -0.41 | -0.53 | -0.50 |
| **RCAR5L** | -0.51 | -0.56 | 0.01 | **0.83** | **0.85** | -0.47 | -0.21 | -0.61 | -0.32 | -0.06 | 0.69 | -0.44 | -0.44 | 0.66 | 0.69 | 0.59 | 0.59 |
| **RCAR6L** | -0.31 | -0.47 | 0.00 | 0.43 | 0.60 | -0.48 | -0.27 | -0.57 | -0.31 | -0.06 | 0.16 | -0.45 | -0.36 | 0.66 | 0.60 | **0.70** | 0.68 |
| **SnRK2.1L** | 0.26 | 0.42 | 0.10 | -0.53 | -0.37 | 0.48 | 0.23 | 0.40 | 0.61 | 0.10 | -0.03 | 0.51 | 0.13 | -0.56 | -0.48 | -0.47 | -0.56 |
| **SnRK2.6L** | 0.20 | 0.12 | 0.00 | -0.24 | -0.14 | -0.04 | 0.07 | -0.08 | 0.32 | -0.34 | 0.04 | 0.09 | 0.36 | -0.06 | -0.16 | 0.15 | 0.14 |
| **PP2C4L** | 0.35 | 0.63 | 0.42 | -0.66 | -0.63 | **0.76** | -0.08 | **0.84** | 0.53 | -0.08 | -0.12 | 0.62 | 0.26 | -**0.74** | -**0.71** | -**0.83** | -**0.85** |
| **PP2C9L** | 0.27 | 0.57 | 0.24 | -0.61 | -0.53 | **0.70** | 0.11 | **0.72** | 0.66 | -0.10 | -0.01 | **0.73** | 0.18 | -0.66 | -0.62 | -**0.70** | -**0.78** |
| **NCED1R** | 0.66 | 0.67 | 0.49 | -0.55 | -0.55 | 0.67 | -0.31 | 0.65 | 0.23 | 0.14 | -0.14 | 0.41 | 0.13 | -0.68 | -0.65 | -**0.76** | -**0.70** |
| **NCED2R** | **1.00** | 0.68 | 0.49 | -0.56 | -0.48 | 0.54 | -0.31 | 0.54 | 0.44 | -0.16 | -0.07 | 0.24 | 0.45 | -0.66 | -**0.71** | -0.50 | -0.43 |
| **ABF1R** | 0.68 | **1.00** | 0.45 | -0.62 | -0.58 | **0.88** | 0.00 | 0.69 | 0.61 | -0.12 | 0.06 | 0.55 | 0.41 | -**0.74** | -**0.76** | -0.61 | -0.62 |
| **ABF2R** | 0.49 | 0.45 | **1.00** | -0.19 | -0.08 | 0.50 | -**0.70** | 0.51 | 0.35 | -0.13 | 0.26 | -0.03 | 0.09 | -0.38 | -0.41 | -0.30 | -0.26 |
| **RCAR5R** | -0.56 | -0.62 | -0.19 | **1.00** | **0.85** | -0.54 | -0.03 | -0.68 | -0.53 | -0.03 | 0.37 | -0.55 | -0.42 | **0.78** | **0.83** | 0.64 | 0.66 |
| **RCAR6R** | -0.48 | -0.58 | -0.08 | **0.85** | **1.00** | -0.48 | -0.23 | -0.67 | -0.33 | -0.16 | 0.48 | -0.44 | -0.37 | 0.66 | **0.70** | 0.64 | 0.64 |
| **SnRK2.1R** | 0.54 | **0.88** | 0.50 | -0.54 | -0.48 | **1.00** | -0.07 | **0.78** | 0.64 | -0.23 | 0.14 | 0.63 | 0.36 | -**0.77** | -**0.72** | -**0.75** | -**0.77** |
| **SnRK2.6R** | -0.31 | 0.00 | -**0.70** | -0.03 | -0.23 | -0.07 | **1.00** | -0.09 | 0.01 | 0.05 | -0.15 | 0.20 | 0.11 | 0.12 | 0.16 | 0.05 | -0.03 |
| **PP2C4R** | 0.54 | 0.69 | 0.51 | -0.68 | -0.67 | **0.78** | -0.09 | **1.00** | 0.63 | -0.22 | -0.09 | 0.47 | 0.48 | -**0.79** | -**0.78** | -**0.70** | -**0.74** |
| **PP2C9R** | 0.44 | 0.61 | 0.35 | -0.53 | -0.33 | 0.64 | 0.01 | 0.63 | **1.00** | -0.38 | 0.24 | 0.53 | 0.43 | -0.67 | -0.66 | -0.45 | -0.56 |
| **Hyd1L** | -0.16 | -0.12 | -0.13 | -0.03 | -0.16 | -0.23 | 0.05 | -0.22 | -0.38 | **1.00** | -0.31 | -0.15 | -0.33 | 0.17 | 0.16 | -0.01 | 0.05 |
| **Hyd1R** | -0.07 | 0.06 | 0.26 | 0.37 | 0.48 | 0.14 | -0.15 | -0.09 | 0.24 | -0.31 | **1.00** | 0.08 | 0.00 | 0.03 | 0.09 | 0.11 | 0.08 |
| **Hyd2L** | 0.24 | 0.55 | -0.03 | -0.55 | -0.44 | 0.63 | 0.20 | 0.47 | 0.53 | -0.15 | 0.08 | **1.00** | 0.39 | -0.63 | -0.60 | -0.63 | -**0.70** |
| **Hyd2R** | 0.45 | 0.41 | 0.09 | -0.42 | -0.37 | 0.36 | 0.11 | 0.48 | 0.43 | -0.33 | 0.00 | 0.39 | **1.00** | -0.55 | -0.61 | -0.26 | -0.30 |
| **SWCmeta** | -0.66 | -**0.74** | -0.38 | **0.78** | 0.66 | -**0.77** | 0.12 | -**0.79** | -0.67 | 0.17 | 0.03 | -0.63 | -0.55 | **1.00** | **0.97** | **0.81** | **0.82** |
| **SWCtrans** | -**0.71** | -**0.76** | -0.41 | **0.83** | **0.70** | -**0.72** | 0.16 | -**0.78** | -0.66 | 0.16 | 0.09 | -0.60 | -0.61 | **0.97** | **1.00** | **0.72** | 0.74 |
| **SWP** | -0.50 | -0.61 | -0.30 | 0.64 | 0.64 | -**0.75** | 0.05 | -**0.70** | -0.45 | -0.01 | 0.11 | -0.63 | -0.26 | **0.81** | **0.72** | **1.00** | **0.98** |
| **RWP** | -0.43 | -0.62 | -0.26 | 0.66 | 0.64 | -**0.77** | -0.03 | -**0.74** | -0.56 | 0.05 | 0.08 | -**0.70** | -0.30 | **0.82** | **0.74** | **0.98** | **1.00** |
