# Supplementary material for: ABA-mediated responses to water deficit separate grapevine genotypes by their genetic background
Source: BMC Plant Biol. 2016 Apr 18;16:91. doi: 10.1186/s12870-016-0778-4 (PMC4836075; doi:10.1186/s12870-016-0778-4)
Supplement: Additional file 8: — Coordinates of variables from the discriminant analysis of gene expression data (F1, F2, Fig. 6) and from principle component analysis of mean transcript abundance and metabolite data (PC1, PC2, Fig. 7). Abbreviation: abscisic acid (ABA), phaseic acid (PA) and dihydrophaseic (DPA) content in shoot (-S) and root (-R) sap; transcript abundance in leaves (-L) and roots (-R). (DOCX 12 kb) [file 12870_2016_778_MOESM8_ESM.docx]

|  | F1 | F2 | PC1 | PC2 |
| --- | --- | --- | --- | --- |
| ABAS | - | - | 0.897 | -0.320 |
| PAS | - | - | 0.710 | -0.192 |
| DPAS | - | - | 0.734 | -0.482 |
| ABAR | - | - | 0.899 | -0.310 |
| PAR | - | - | 0.764 | -0.179 |
| DPAR | - | - | 0.880 | -0.297 |
| NCED1L | -0.417 | -0.130 | 0.887 | -0.202 |
| NCED2L | 0.488 | -0.108 | -0.234 | 0.616 |
| ABF1L | -0.454 | -0.124 | 0.902 | -0.188 |
| ABF2L | -0.460 | 0.582 | 0.596 | -0.346 |
| Hyd1L | -0.390 | -0.042 | 0.001 | -0.457 |
| Hyd2L | -0.016 | -0.323 | 0.696 | 0.238 |
| RCAR5L | 0.037 | 0.235 | -0.672 | -0.303 |
| RCAR6L | 0.399 | 0.368 | -0.666 | 0.050 |
| SnRK2.1L | -0.078 | -0.323 | 0.558 | 0.254 |
| SnRK2.6L | 0.850 | 0.164 | -0.166 | 0.750 |
| PP2C4L | -0.318 | 0.061 | 0.929 | -0.059 |
| PP2C9L | -0.229 | -0.127 | 0.861 | 0.042 |
| NCED1R | -0.316 | 0.054 | 0.778 | -0.087 |
| NCED2R | 0.149 | 0.090 | 0.477 | 0.527 |
| ABF1R | 0.008 | -0.117 | 0.726 | 0.437 |
| ABF2R | -0.119 | 0.518 | 0.396 | 0.042 |
| Hyd1R | 0.073 | 0.046 | -0.140 | 0.080 |
| Hyd2R | 0.235 | -0.099 | 0.314 | 0.620 |
| RCAR5R | -0.065 | 0.061 | -0.753 | -0.386 |
| RCAR6R | 0.037 | 0.126 | -0.723 | -0.205 |
| SnRK2.1R | -0.113 | -0.079 | 0.809 | 0.266 |
| SnRK2.6R | 0.132 | -0.690 | -0.023 | 0.125 |
| PP2C4R | -0.172 | 0.049 | 0.859 | 0.172 |
| PP2C9R | 0.179 | -0.038 | 0.559 | 0.570 |
