# Supplementary material for: ABA-mediated responses to water deficit separate grapevine genotypes by their genetic background
Source: BMC Plant Biol. 2016 Apr 18;16:91. doi: 10.1186/s12870-016-0778-4 (PMC4836075; doi:10.1186/s12870-016-0778-4)
Supplement: Additional file 9: — Fresh biomass, leaf area and soil water content for nine grapevine genotypes. Values represent mean ± standard deviation (n = 24, except for soil water content on day 1 and 4, n = 6). Values among genotypes with the same letter are not statistical different (one way ANOVA, p-value is presented in bottom line). (DOCX 20 kb) [file 12870_2016_778_MOESM9_ESM.docx]

|  | **Leaves (g)** | | | | **Stem (g)** | | | | **Trunk (g)** | | | | **Roots (g)** | | | | **Total biomass (g)** | | | |
| --- | --- | --- | --- | --- | --- | --- | --- | --- | --- | --- | --- | --- | --- | --- | --- | --- | --- | --- | --- | --- |
|  | mean |  | sd |  | mean |  | sd |  | mean |  | sd |  | mean |  | sd |  | mean |  | sd |  |
| **RGM** | 4.53 | ± | 0.65 | e | 5.85 | ± | 1.23 | abcd | 5.59 | ± | 0.57 | de | 10.37 | ± | 4.29 | abc | 26.35 | ± | 5.22 | bc |
| **101-14 Mgt** | 4.68 | ± | 0.49 | de | 6.73 | ± | 1.57 | a | 4.85 | ± | 0.88 | e | 9.37 | ± | 3.16 | abc | 25.65 | ± | 4.01 | bc |
| **SO4** | 4.21 | ± | 0.39 | e | 5.11 | ± | 0.69 | de | 7.00 | ± | 1.62 | a | 11.59 | ± | 3.48 | ab | 27.94 | ± | 4.07 | ab |
| **161-49 C** | 4.57 | ± | 0.44 | e | 5.37 | ± | 1.43 | cd | 6.73 | ± | 0.69 | ab | 9.68 | ± | 3.07 | abc | 26.35 | ± | 3.61 | bc |
| **41B Mgt** | 5.79 | ± | 0.42 | a | 5.02 | ± | 0.97 | de | 6.03 | ± | 0.92 | bcd | 8.32 | ± | 3.61 | c | 25.17 | ± | 4.20 | bc |
| **110R** | 5.23 | ± | 0.45 | bc | 5.67 | ± | 1.19 | bcd | 5.97 | ± | 1.26 | bcd | 7.97 | ± | 4.12 | c | 24.85 | ± | 5.07 | bc |
| **140Ru** | 5.06 | ± | 0.54 | cd | 4.14 | ± | 0.69 | e | 5.80 | ± | 1.06 | cd | 8.46 | ± | 3.09 | bc | 23.47 | ± | 3.53 | c |
| **Syrah** | 5.26 | ± | 0.57 | bc | 6.60 | ± | 1.25 | ab | 6.67 | ± | 1.28 | abc | 9.18 | ± | 4.81 | bc | 27.73 | ± | 5.68 | ab |
| **Grenache** | 5.59 | ± | 0.91 | ab | 6.28 | ± | 1.46 | abc | 6.07 | ± | 1.29 | bcd | 12.43 | ± | 4.30 | a | 30.39 | ± | 6.21 | a |
| **ANOVA p-value** | < 0.0001 | | | | < 0.0001 | | | | < 0.0001 | | | | 0.0003 | | | | < 0.0001 | | | |
|  |  |  |  |  |  |  |  |  |  |  |  |  |  |  |  |  |  |  |  |  |
|  | **Leaf area (cm²)** | | | | **Leaf area/Root biomass (cm² g^-1^)** | | | | **Leaves/root biomass (g g^-1^)** | | | | **SWC day 1 (g g^-1^)** | | | | **SWC day 4 (g g^-1^)** | | | |
|  | mean |  | sd |  | mean |  | sd |  | mean |  | sd |  | mean |  | sd |  | mean |  | sd |  |
| **RGM** | 443.0 | ± | 50.14 | a | 49.28 | ± | 18.00 | abc | 0.50 | ± | 0.20 | bcd | 0.13 | ± | 0.009 | ab | 0.028 | ± | 0.023 | a |
| **101-14 Mgt** | 414.4 | ± | 34.64 | bc | 50.38 | ± | 20.68 | abc | 0.56 | ± | 0.23 | bcd | 0.12 | ± | 0.009 | abc | 0.013 | ± | 0.009 | ab |
| **SO4** | 404.5 | ± | 33.54 | bc | 37.89 | ± | 11.10 | c | 0.39 | ± | 0.12 | d | 0.13 | ± | 0.009 | a | 0.019 | ± | 0.002 | ab |
| **161-49 C** | 391.8 | ± | 27.70 | c | 44.43 | ± | 14.01 | bc | 0.51 | ± | 0.15 | bcd | 0.13 | ± | 0.008 | ab | 0.021 | ± | 0.008 | ab |
| **41B Mgt** | 417.5 | ± | 24.35 | bc | 58.04 | ± | 20.94 | ab | 0.80 | ± | 0.28 | a | 0.10 | ± | 0.009 | d | 0.011 | ± | 0.001 | b |
| **110R** | 413.5 | ± | 26.90 | bc | 63.39 | ± | 27.56 | a | 0.80 | ± | 0.34 | a | 0.12 | ± | 0.009 | abc | 0.011 | ± | 0.004 | b |
| **140Ru** | 408.8 | ± | 27.43 | bc | 55.86 | ± | 23.80 | ab | 0.68 | ± | 0.26 | abc | 0.11 | ± | 0.007 | cd | 0.007 | ± | 0.003 | b |
| **Syrah** | 423.0 | ± | 21.79 | ab | 56.24 | ± | 22.82 | ab | 0.69 | ± | 0.27 | ab | 0.11 | ± | 0.007 | bcd | 0.013 | ± | 0.003 | ab |
| **Grenache** | 414.1 | ± | 28.05 | bc | 38.16 | ± | 15.92 | c | 0.48 | ± | 0.13 | cd | 0.11 | ± | 0.010 | cd | 0.015 | ± | 0.004 | ab |
| **ANOVA p-value** | < 0.0001 | | | | < 0.0001 | | | | < 0.0001 | | | | < 0.0001 | | | | 0.011 | | | |
