# Supplementary material for: ABA-mediated responses to water deficit separate grapevine genotypes by their genetic background
Source: BMC Plant Biol. 2016 Apr 18;16:91. doi: 10.1186/s12870-016-0778-4 (PMC4836075; doi:10.1186/s12870-016-0778-4)
Supplement: Additional file 10: — Primer pair sequences used in this study. Accessions number from Gramene: http://www.gramene.org. (DOCX 14 kb) [file 12870_2016_778_MOESM10_ESM.docx]

|  | Forward primer (5' to 3') | Reverse primer (5' to 3') | Gene ID | Closest *Arabidopsis thaliana* ortholog |
| --- | --- | --- | --- | --- |
| VviNCED1 | TTCCCTCACGAGTTCCCTATG | TCCTCTGCAATCTGACACCAAG | [VIT_19s0093g00550](http://ensembl.gramene.org/Vitis_vinifera/Gene/Summary?db=core;g=VIT_19s0093g00550;r=19:17645348-17647649;tl=B5uktnJSlvc1vEKY-58310-17795883) | AtNCED3 |
| VviNCED2 | AGTTCCATACGGGTTTCATGGG | CCATTTTCCAAATCCAGGGTGT | [VIT_10s0003g03750](http://ensembl.gramene.org/Vitis_vinifera/Gene/Summary?db=core;g=VIT_10s0003g03750;r=19:17645348-17647649;tl=mpFPP8cDKC5VtgYI-58382-17796109) | AtNCED5 |
| VviHyd1 | CTTCCCCTGACTTCGTTTT | CGTACAAAATCAAAGTAGGGATA | [VIT_18s0001g10500](http://ensembl.gramene.org/Vitis_vinifera/Gene/Summary?db=core;g=VIT_18s0001g10500;r=19:17645348-17647649;tl=Cv2h2ba697ikwll7-58387-17796127) | AtABA 8' hydroxylase 4 |
| VviHyd2 | TGGGCACACAGAATGGTATT | TGATTGGTGGCACTGAGAG | [VIT_02s0087g00710](http://ensembl.gramene.org/Vitis_vinifera/Gene/Summary?db=core;g=VIT_02s0087g00710;r=19:17645348-17647649;tl=1gTXFSeRp3PZcgWw-58400-17796173) | AtABA 8' hydroxylase 1 |
| VviRCAR5 | GTTTTTGTCGACACCATCGTT | TCATGACGATGACCTCTTGC | [VIT_08s0058g00470](http://ensembl.gramene.org/Vitis_vinifera/Gene/Summary?db=core;g=VIT_08s0058g00470;r=19:17645348-17647649;tl=dxgcCpxlvqwJAhSo-58409-17796220) | AtPYL4 |
| VviRCAR6 | CGGGTAACACGAAGGAGGA | GGGGTGCAATAATCTAAAAGAG | VIT_13s0067g01940 | AtPYL4 |
| VviPP2C4 | TGGGCTTTGGGATGTTATGT | TGTGCAGGAGTCTCATCAGC | [VIT_11s0016g03180](http://ensembl.gramene.org/Vitis_vinifera/Gene/Summary?db=core;g=VIT_11s0016g03180;r=19:17645348-17647649;tl=tZBpJDlrfxTyQOWu-58412-17796356) | AtABI1 |
| VviPP2C9 | TTAAAGCCCTTCGTGAGCTG | GACACCACGTCCCACAGAC | [VIT_06s0004g05460](http://ensembl.gramene.org/Vitis_vinifera/Gene/Summary?db=core;g=VIT_06s0004g05460;r=19:17645348-17647649;tl=E5Ps2foaABnoYvd7-58413-17796376) | AtPP2C3 |
| VviSnRK2.1 | TTTTTGTGGCAAACCCAGAT | CAGCTTCCTCCATCCATCAT | [VIT_18s0001g06310](http://ensembl.gramene.org/Vitis_vinifera/Gene/Summary?db=core;g=VIT_18s0001g06310;r=19:17645348-17647649;tl=1qkfoUAD7kLnvgug-58414-17796386) | AtSnRK2C/AtSnRK2.8 |
| VviSnRK2.6 | CACCAACCCACCTTGCTATT | AAACGTGCCTCATCCTCACT | [VIT_03s0063g0](http://ensembl.gramene.org/Vitis_vinifera/Gene/Summary?db=core;g=VIT_18s0001g06310;r=19:17645348-17647649;tl=1qkfoUAD7kLnvgug-58414-17796386)1080 | AtOST1/AtSnRK2.6 |
| VviABF1 | TGCCAATCAGTCATCAGA | CCTTCCTCTTATACCTCCAT | VIT_18s0001g10450 | AtABRE-binding factor 2/AtbZIP36 |
| VviABF2 | GGCACCCAGGCTAGTTAA | GCAGAGTACACGCTAGATTG | VIT_03s0063g00310 | AtABRE-binding factor 3/AtbZIP37 |
| VviActin | CTTGCATCCCTCAGCACCTT | TCCTGTGGACAATGGATGGA | [VIT_04s0044g00580](http://ensembl.gramene.org/Vitis_vinifera/Gene/Summary?db=core;g=VIT_04s0044g00580;r=19:17645348-17647649;tl=115j2j4TVBA7IoC7-58433-17796681) | AtActin-7 |
| VviEF1γ | CAAGAGAAACAATCCCTAGCTG | TCAATCTGTCTAGGAAAGGAAG | [VIT_12s0035g01130](http://ensembl.gramene.org/Vitis_vinifera/Gene/Summary?db=core;g=VIT_12s0035g01130;r=19:17645348-17647649;tl=KpEN9LUgOvjSCiXd-58434-17796690) | AtEF-1-gamma 1 |
| VviGAPDH | CCACAGACTTCATCGGTGACA | TTCTCGTTGAGGGCTATTCCA | [VIT_17s0000g10430](http://ensembl.gramene.org/Vitis_vinifera/Gene/Summary?db=core;g=VIT_17s0000g10430;r=19:17645348-17647649;tl=l1gXd11TtL6vmdDQ-58435-17796695) | AtGAPC2 |
